# Cell-autonomous light sensitivity via Opsin3 regulates fuel utilization in brown adipocytes

**DOI:** 10.1371/journal.pbio.3000630

**Published:** 2020-02-10

**Authors:** Mari Sato, Tadataka Tsuji, Kunyan Yang, Xiaozhi Ren, Jonathan M. Dreyfuss, Tian Lian Huang, Chih-Hao Wang, Farnaz Shamsi, Luiz O. Leiria, Matthew D. Lynes, King-Wai Yau, Yu-Hua Tseng

**Affiliations:** 1 Section on Integrative Physiology and Metabolism, Joslin Diabetes Center, Harvard Medical School, Boston, Massachusetts, United States of America; 2 Oral Biochemistry and Molecular Biology, Graduate School of Dental Medicine, Hokkaido University, Sapporo, Japan; 3 JST, PRESTO, Kawaguchi, Japan; 4 Solomon H. Snyder Department of Neuroscience, Johns Hopkins University School of Medicine, Baltimore, Maryland, United States of America; 5 Bioinformatics and Biostatistics Core, Joslin Diabetes Center, Harvard Medical School, Boston, Massachusetts, United States of America; 6 Department of Pharmacology, Ribeirao Preto Medical School, University of São Paulo, Ribeirão Preto, Brazil; 7 Center of Research of Inflammatory Diseases, Ribeirao Preto Medical School, University of São Paulo, Ribeirão Preto, Brazil; 8 Harvard Stem Cell Institute, Harvard University, Cambridge, Massachusetts, United States of America; University of Pennsylvania, UNITED STATES

## Abstract

Opsin3 (Opn3) is a transmembrane heptahelical G protein–coupled receptor (GPCR) with the potential to produce a nonvisual photoreceptive effect. Interestingly, anatomical profiling of GPCRs reveals that *Opn3* mRNA is highly expressed in adipose tissue. The photosensitive functions of Opn3 in mammals are poorly understood, and whether Opn3 has a role in fat is entirely unknown. In this study, we found that *Opn3*-knockout (*Opn3*-KO) mice were prone to diet-induced obesity and insulin resistance. At the cellular level, *Opn3*-KO brown adipocytes cultured in darkness had decreased glucose uptake and lower nutrient-induced mitochondrial respiration than wild-type (WT) cells. Light exposure promoted mitochondrial activity and glucose uptake in WT adipocytes but not in *Opn3*-KO cells. Brown adipocytes carrying a defective mutation in Opn3’s putative G protein–binding domain also exhibited a reduction in glucose uptake and mitochondrial respiration in darkness. Using RNA-sequencing, we identified several novel light-sensitive and Opn3-dependent molecular signatures in brown adipocytes. Importantly, direct exposure of brown adipose tissue (BAT) to light in living mice significantly enhanced thermogenic capacity of BAT, and this effect was diminished in *Opn3*-KO animals. These results uncover a previously unrecognized cell-autonomous, light-sensing mechanism in brown adipocytes via Opn3-GPCR signaling that can regulate fuel metabolism and mitochondrial respiration. Our work also provides a molecular basis for developing light-based treatments for obesity and its related metabolic disorders.

## Abstract 2

Brown adipose tissue plays a pivotal role in energy homeostasis and serves as a metabolic sink for glucose and fatty acid. This study demonstrates a novel light-sensing mechanism in mice via the photoreceptor Opsin3 that regulates fuel utilization and mitochondrial activity of brown adipocytes.

## Introduction

Obesity alters adipose tissue metabolic and endocrine functions and leads to the development of several common medical conditions, such as type 2 diabetes mellitus, cardiovascular diseases, nonalcoholic fatty liver, and even cancer [1]. Mammals have two functionally distinct types of adipose tissue. White adipose tissue (WAT) contains cells with a single lipid droplet and is responsible for fuel storage [2]. Brown and related beige adipose tissue contains cells with multilocular lipid droplets and is specialized for energy expenditure by releasing heat [3]. Because altering the energy balance of adipose tissue contributes to the development and progression of metabolic diseases, increasing our understanding of the regulation of adipose tissues may uncover potential approaches for the treatment of obesity and related metabolic disorders.

Brown adipose tissue (BAT) exists mainly in the interscapular region in rodents. Although BAT is also found in the same region in human infants, it gradually disappears with age, and in adults, active BAT is predominantly located in the deep neck and cervical supraclavicular regions [4–6]. BAT combusts glucose and fatty acid and dissipates the energy as heat via mitochondrial expression of uncoupling protein-1 (UCP1) [3,7]. Because BAT activation contributes to the improvement of whole-body glucose homeostasis and insulin sensitivity in both rodents and humans [8–10], ways to increase the activity of BAT hold great potential for the treatment and prevention of obesity and its sequelae. Cold exposure is a well-known external stimulus to activate BAT [5,11], but little is known about other environmental or internal cues that could activate BAT.

Opsin3 (Opn3) was cloned in 1999 and found to be widely expressed in the mouse brain [12]. A decade later, Opn3 was found to be also highly expressed in adipose tissue [13]. Interestingly, distant homologs of Opn3 are capable of mediating direct photoreception in various nonvisual tissues by activating G protein–coupled receptor (GPCR) signaling in a light-dependent manner [14–16]. These lines of evidence prompted us to ask whether adipose tissue can indeed sense light directly via Opn3-GPCR signaling and, if so, what the metabolic consequences of these signaling pathways are. Phototherapy has been proposed as a way to reduce body fat and improve diabetic wound healing [17–19]. However, the efficacy of such therapy has not been thoroughly evaluated, and the underlying mechanisms are entirely unknown. Our findings raise the possibility that Opn3 is a potential target of phototherapy in adipose tissues.

## Results

### Mice lacking *Opn3* become obese and insulin resistant upon high-fat feeding

**Fig 1:**
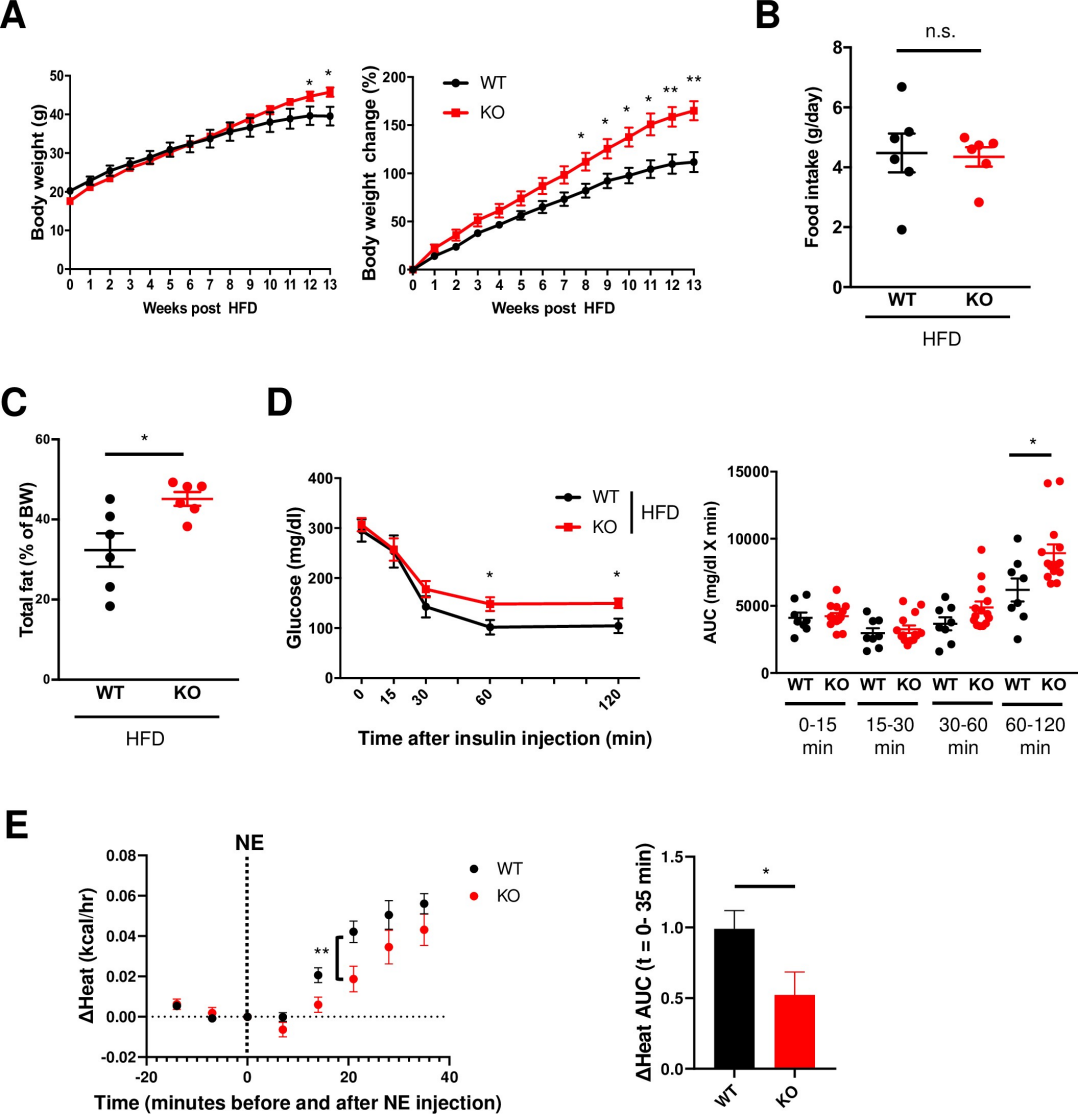
*Opn3*-KO mice display obese and insulin-resistant phenotypes upon high-fat feeding. (A) *Opn3*-KO and littermate WT mice at the age of 4–5 weeks were fed with an HFD for 13 weeks. Left: BW was measured on a weekly basis. Right: The percent change of BW (*n* = 8–13). (B) Average daily food intake calculated after 13 weeks of HFD (*n* = 6). (C) Total fat volume measured by DEXA scan after 13 weeks of HFD and normalized to BW (*n* = 6). (D) Left: ITT performed after 12 weeks of HFD. Mice were fasted for 6 hours, followed by an IP injection of insulin. Blood glucose levels were determined at the indicated time after injection. Right: AUC for the ITT was calculated for the respective time interval (*n* = 8–13). (E) Left: ΔHeat (increase in energy expenditure) measured by CLAMS for 35 minutes in WT and *Opn3*-KO mice after IP injection of NE (*n* = 6). Right: AUC quantification of ΔHeat is shown. WT and *Opn3*-KO mice were fed with normal chow diet, and 15-week-old mice were utilized. Data are represented as means ± SEM. The *p*-values were determined by unpaired *t* test ([A–E], right) and two-way repeated measures ANOVA followed by Bonferroni’s test ([E], left). *p < 0.05, **p < 0.01. The data for this figure can be found in the Dryad repository: https://doi.org/10.5061/dryad.p5hqbzkkv [70]. AUC, area under the curve; BW, body weight; CLAMS, Comprehensive Lab Animal Monitoring System; DEXA, dual-energy X-ray absorptiometry; HFD, high-fat diet; IP, intraperitoneal; ITT, insulin-tolerance test; KO, knockout; NE, norepinephrine; n.s., not significant; *Opn3*, Opsin3; WT, wild-type.

The global *Opn3*-knockout (KO) mice display normal physiology and no obvious visual defect [15]. There was no difference in body weight between adult wild-type (WT) and *Opn3-*KO mice on a normal chow diet (NCD) (S1A Fig), suggesting that Opn3 does not impact adipose development or energy metabolism under normal conditions. However, when challenged with a high-fat diet (HFD), *Opn3*-KO mice gained more weight than littermate WT animals (Fig 1A), despite equal food intake (Fig 1B). This was associated with an increase in fat mass in HFD-fed *Opn3*-KO mice compared with HFD-fed WT animals (Fig 1C). Moreover, HFD-fed *Opn3*-KO mice were more insulin resistant compared with HFD-fed WT mice (Fig 1D), although there was no difference in glucose tolerance between HFD-fed WT and *Opn3*-KO animals (S1B Fig). Thus, *Opn3*-KO mice were prone to diet-induced obesity and insulin resistance. In addition, *Opn3*-KO mice displayed impaired maximum thermogenic capacity, with reduced heat production (“Heat”) and reduced oxygen consumption (“VO_2_”) in response to norepinephrine (NE) treatment compared with WT mice (Fig 1E, S1C Fig). Taken together, these data suggest that Opn3 may directly contribute to whole-body metabolism, probably through regulation of fuel utilization for adaptation to metabolic challenges.

### Opn3 is important for fuel metabolism and mitochondrial respiration in brown adipose cells

**Fig 2:**
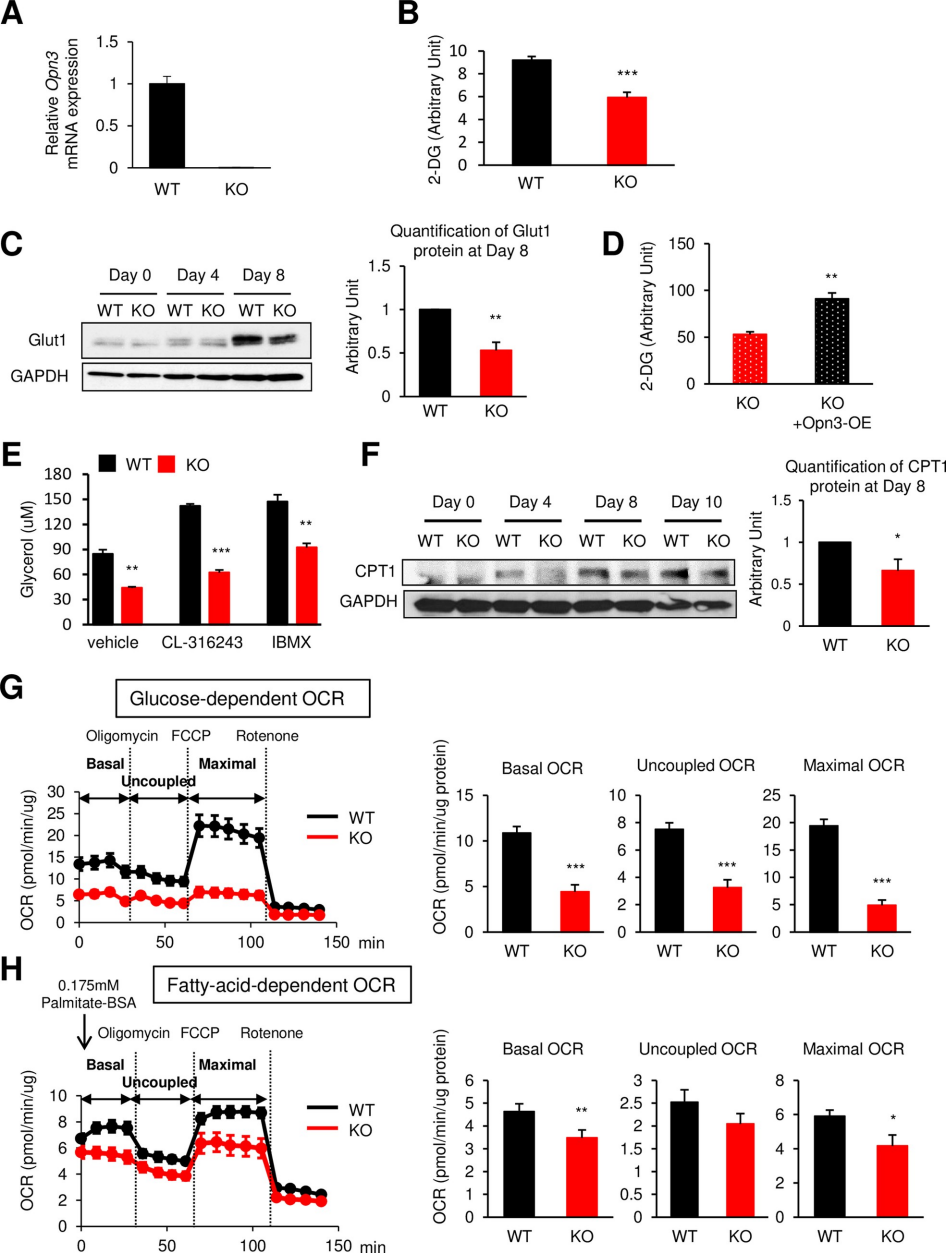
*Opn3*-KO brown adipocytes display impaired fuel intake and usage. (A) *Opn3* mRNA expression in WT and *Opn3*-KO brown preadipocytes (*n* = 4). (B) Basal glucose uptake was measured by using 2-DG in differentiated WT and *Opn3*-KO brown adipocytes (*n* = 4). The experiment was repeated independently four times. (C) Left: Western blot analysis of Glut1 protein in WT and *Opn3*-KO brown adipocytes at day 0, 4, and 8 of differentiation. Right: Quantification of Glut1 protein at day 8 (*n* = 3). This experiment was repeated three times with similar results. (D) Basal glucose uptake in differentiated *Opn3*-KO brown adipocytes and *Opn3*-KO cells with overexpression of *Opn3* (KO + Opn3-OE) (*n* = 6). The experiment was performed in three independent experiments. (E) Lipolysis was measured in differentiated WT and *Opn3*-KO brown adipocytes stimulated with vehicle, CL-316,243 (5 μM), or IBMX (100 μM) (*n* = 3). The experiment was repeated independently two times. (F) Left: Western blot analysis of CPT1 in WT and *Opn3*-KO brown preadipocytes at day 0, 4, 8, and 10 of differentiation. Right: Quantification of CPT1 protein at day 8 (*n* = 4). The experiment on day 8 was repeated four times with similar results. (G) Left: Glucose-dependent OCR in differentiated WT and *Opn3*-KO brown adipocytes. FCCP (*n* = 11). Right: Quantification of OCR (*n* = 11). The experiment was repeated independently five times. (H) Left: Fatty acid–dependent OCR in differentiated WT and *Opn3*-KO brown adipocytes (*n* = 7). Right: Quantification of OCR (*n* = 7). The experiment was repeated independently three times. In all of the above experiments, cells were cultured and differentiated under the normal dark condition in a CO_2_ incubator. The values denote the mean ± SEM, and comparisons were made by Student *t* test. *p < 0.05; **p < 0.01; ***p < 0.001. The data for this figure can be found in the Dryad repository: https://doi.org/10.5061/dryad.p5hqbzkkv [70]. 2-DG, 2-deoxy-D-glucose; CPT1, carnitine palmitoyltransferase 1; BSA, bovine serum albumin; FCCP, carbonyl cyanide 4-(trifluoromethoxy) phenylhydrazone; GAPDH, Glyceraldehyde-3-phosphate dehydrogenase; Glut1, Glucose transporter 1; IBMX, 3-isobutyl-1-methylxanthine; KO, knockout; OCR, oxygen consumption rate; Opn3, Opsin3; Opn3-OE, overexpression of *Opn3*; WT, wild-type.

In recent years, BAT has been demonstrated to play a pivotal role in energy expenditure and systemic metabolism [20,21]. Because *Opn3*-KO mice showed reduced maximal thermogenic capacity, we examined whether Opn3 is involved in cellular nutrient utilization in brown adipocytes in vitro. Analysis of several *opsin* genes in WT brown adipocyte precursors (preadipocytes) showed high expression of *Opn3* compared with other members of the opsin family (S2A Fig), and the expression continued to increase during adipogenic differentiation (S2B Fig). Moreover, not only murine but also human brown adipocytes expressed high levels of *Opn3* mRNA (S2C Fig). We found that BAT from adult *Opn3*-KO mice lacked *Opn3* mRNA expression, with no compensatory increase in the expressions of other *opsins* (S1D Fig). For a direct comparison, we isolated preadipocytes from BAT of WT and *Opn3*-KO mice in parallel and immortalized them as described previously [22]. Cells were cultured and differentiated for 8 days in darkness in a CO_2_ incubator. *Opn3* mRNA was undetectable in *Opn3*-KO brown preadipocytes (Fig 2A), but these cells were nonetheless able to differentiate normally in response to adipogenic induction, with the levels of *adipocyte protein 2* (*AP2*) mRNA and protein, peroxisome proliferator–activated receptor g (PPARg) protein, and lipid accumulation unaffected (S2D–S2G Fig). However, mRNA and protein expression of the BAT-specific marker *Ucp1* was significantly lower in *Opn3*-KO cells (S2H Fig). These results were replicated in two additional lines of immortalized *Opn3*-KO brown preadipocytes (S2I–S2K Fig).

Because BAT controls whole-body metabolism by utilizing glucose and fatty acid as fuel sources [8,9], we measured glucose and fatty acid uptake as well as mitochondrial oxygen consumption rate (OCR) in WT and *Opn3*-KO brown adipocytes. Basal glucose uptake was significantly decreased in *Opn3*-KO brown adipocytes compared with WT cells (Fig 2B), presumably because of a reduction of the glucose transporter 1 (Glut1) protein in the KO cells (Fig 2C). Similar levels of reduction in glucose uptake were observed in the other two immortalized *Opn3*-KO cell lines compared with WT cells (S2L Fig). Genetic rescue of Opn3 in *Opn3*-KO brown preadipocytes by transfecting the KO cells with a plasmid expressing Opn3 cDNA resulted in overexpression of Opn3 (S2M Fig, left). This led to elevated levels of *Ucp1* mRNA expression (S2M Fig, right) and was able to rescue the defect in glucose uptake in *Opn3*-KO cells (Fig 2D), suggesting that the decreased metabolic function of *Opn3*-KO brown adipocytes was indeed due to the lack of Opn3.

In addition to reduced glucose uptake, we found that the *Opn3*-KO brown adipocytes also displayed significantly reduced lipolytic rates in basal condition and in response to the β3-adrenergic receptor agonists CL-316,243 and 3-isobutyl-1-methylxanthine (IBMX) (Fig 2E). This lower lipolytic rate was likely due to a reduced level of the adipose tissue triglyceride lipase (ATGL) protein but not the hormone-sensitive lipase (HSL), which appeared to be unchanged in *Opn3*-KO cells (S2N Fig). Expression of carnitine palmitoyltransferase 1 (CPT1) protein, which is the rate-limiting enzyme for transporting fatty acid into mitochondria, was also reduced by 34% ± 13.48% in *Opn3*-KO brown adipocytes compared with WT cells (Fig 2F). Somewhat paradoxically, fatty acid uptake into the cells was slightly increased in the *Opn3*-KO cells (S2O Fig). Overall, these data suggest that deletion of Opn3 in brown adipocytes alters their nutrient utilization.

Next, we analyzed glucose- or fatty acid–dependent mitochondrial OCR by treating the differentiated brown adipocytes with oligomycin, an inhibitor of ATP synthase, and carbonyl cyanide 4-(trifluoromethoxy) phenylhydrazone (FCCP), a mitochondrial uncoupler. Before the reagents were added, basal OCR was measured, and the maximal OCR was then measured by exposure to FCCP. As a consequence of a decreased capacity to shuttle nutrients into mitochondria, *Opn3*-KO cells displayed a blunted mitochondrial respiration in the presence of glucose or fatty acid as the fuel source compared with WT cells (Fig 2G and 2H). Moreover, mitochondrial DNA copy number, which was determined by expression of the mitochondrially encoded mitochondrial NADH dehydrogenase subunit 1 (ND1) and 6 (ND6) normalized to nuclear DNA, as well as the activity of cytochrome c oxidase in the mitochondria were both significantly lower in *Opn3*-KO compared with control brown adipocytes (S2P and S2Q Fig). These data highlight a critical role of Opn3 in the regulation of nutrient metabolism and mitochondrial respiration in brown adipocytes under the normal dark condition.

### Light stimulation through Opn3 activates fuel metabolism and mitochondrial respiration in brown adipocytes

**Fig 3:**
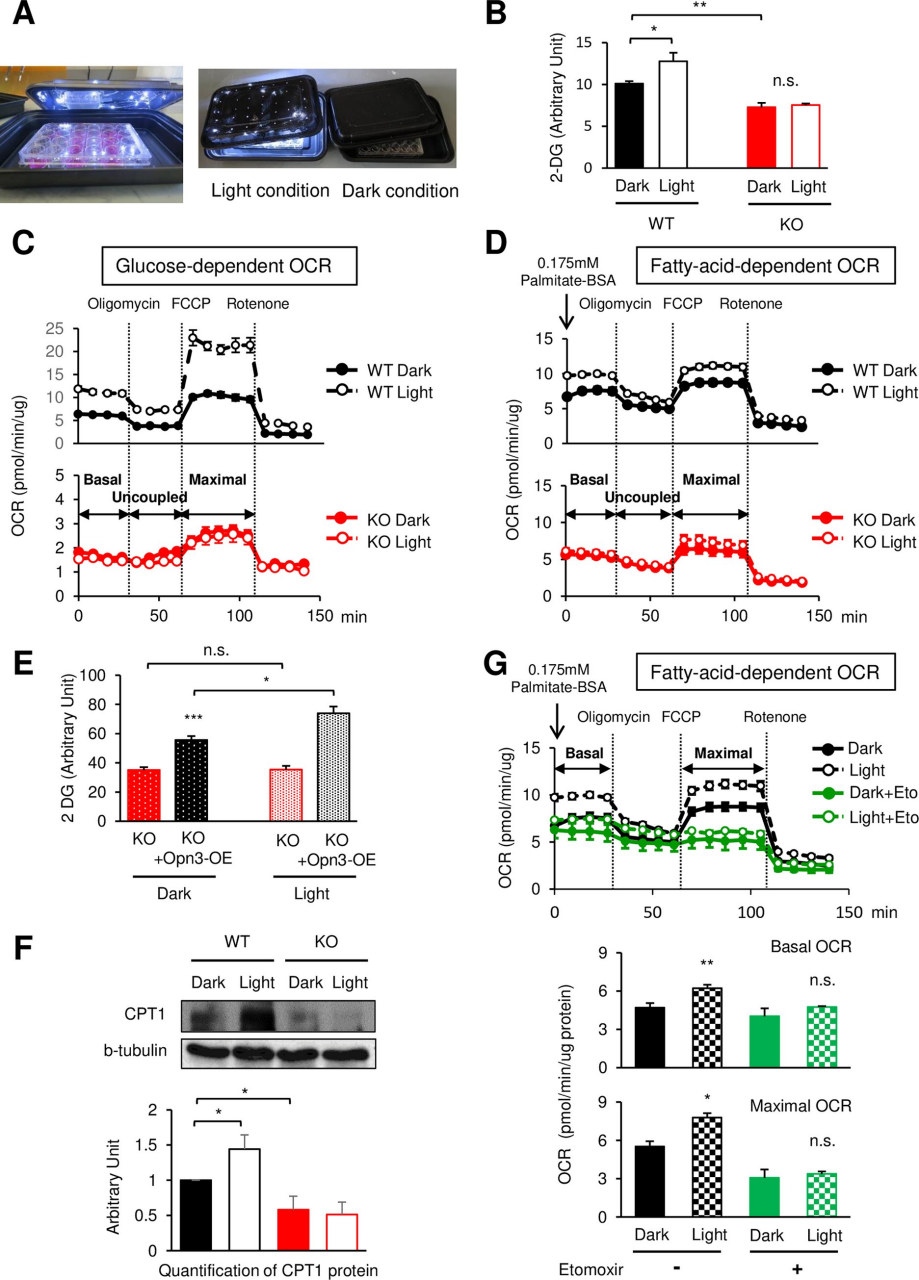
Light stimulation via Opn3 enhances fuel intake and usage in brown adipocytes. (A) Crafted black box with or without white LED light. (B) Glucose uptake of differentiated WT and *Opn3*-KO brown adipocytes with or without light stimulation (*n* = 4–5). The experiment was repeated independently three times. (C) Glucose-dependent OCR in WT and *Opn3*-KO brown adipocytes with or without light stimulation. FCCP (*n* = 10–11). The experiment was repeated independently three times. (D) Fatty acid–dependent OCR in WT and *Opn3*-KO brown adipocytes with or without light stimulation (*n* = 7). The experiment was repeated independently three times. (E) Glucose uptake of *Opn3*-KO and KO + Opn3-OE brown adipocytes with or without light (*n* = 5–6). The experiment was performed in three biological independent experiments. (F) Upper: Western blot analysis of CPT1 protein level in WT and *Opn3*-KO brown adipocytes differentiated in the light or dark condition. Lower: Quantification of CPT1 protein (*n* = 4). This experiment was repeated four times with similar results. (G) Upper: Fatty acid–dependent OCR was measured in the presence or absence of CPT1 inhibitor Eto in brown adipocytes differentiated in the light or dark condition (*n* = 3–7). Lower: Quantification of OCR (*n* = 3–7). The experiment was repeated independently three times. The values denote the mean ± SEM, and comparisons were made by Student *t* test. *p < 0.05; **p < 0.01; ***p < 0.001. The data for this figure can be found in the Dryad repository: https://doi.org/10.5061/dryad.p5hqbzkkv [70]. 2-DG, 2-deoxy-D-glucose; BSA, Bovine serum albumin; CPT1, carnitine palmitoyltransferase 1; Eto, etomoxir; FCCP, carbonyl cyanide 4-(trifluoromethoxy) phenylhydrazone; KO, knockout; LED, light-emitting diode; n.s., not significant; OCR, oxygen consumption rate; Opn3, Opsin3; Opn3-OE, overexpression of *Opn3*; WT, wild-type.

Because Opn3 contains sequence motifs characteristic of a light sensor [14,23,24], we hypothesized that light could regulate the metabolic function of brown adipocytes via Opn3 in a cell-autonomous manner. To test this hypothesis, we seeded brown preadipocytes in culture plates inside black boxes equipped with light-emitting diodes (LEDs) capable of emitting 450–650 nm light (Fig 3A, S3A Fig). The boxes with and without LED lights were kept in a cell culture incubator for 8 days to allow adipogenic differentiation. We also made sure that there was sufficient airflow through the black boxes in the incubator and that the LED light did not change the temperature of the culture media when turned on (S3B Fig). *Opn3* mRNA expression was not affected by light exposure in both differentiated WT and *Opn3*-KO brown adipocytes (S3C Fig). Light exposure significantly increased glucose uptake and glucose-dependent mitochondrial respiration in brown adipocytes (Fig 3B and 3C, and S3D Fig). Although light did not change lipolysis, expression of ATGL protein, mitochondrial number, or cytochrome c oxidase activity of the cells (S3E–S3H Fig), it noticeably increased both basal and maximal VO_2_ when palmitate was provided as a fuel (Fig 3D, S3I Fig). Importantly, the rates of glucose uptake and mitochondrial respiration in *Opn3*-KO cells, already lower in the dark condition compared with WT cells, were almost completely blunted in response to light stimulation (Fig 3B–3D, S3D and S3I Fig). Moreover, reexpression of Opn3 restored the impaired glucose uptake in both dark and light conditions in *Opn3*-KO cells (Fig 3E). At the molecular level, the expression of CPT1 protein was significantly enhanced by light exposure in WT cells, and both basal and light-induced CPT1 protein expressions were diminished in *Opn3*-KO cells (Fig 3F). In addition, the CPT1 inhibitor etomoxir abolished light-induced, fatty acid–dependent VO_2_ (Fig 3G). These results suggest that light stimulation through Opn3 can regulate glucose and fatty acid metabolism in brown adipose cells.

Light stimulation is well-known as an essential trigger for the activation of the central clock [25], whereas a food cue is the main inducer for activating the peripheral clock [26]. Clock genes, controlled by both central and peripheral clocks, regulate whole-body metabolism [27]. Although light can potentially trigger the peripheral clock independently of the central clock [28] in vivo, we nonetheless found that light did not change rhythmic expression of the circadian genes—namely, *Period-2* (*Per2*), *Period-3* (*Per3*), *Clock*, and *BMAL1*—in brown adipocytes (S3J Fig), whereas expression of *Period-1* (*Per1*), *Cryptochrome-1* (*Cry1*), and *Cryptochrome-2* (*Cry2*) mRNA was slightly altered after 24 hours of light exposure. These results suggest that light stimulation via Opn3 controls fuel utilization in brown adipocytes in a clock-independent manner.

### Opn3 regulates cellular metabolism of brown adipocytes via a GPCR-mediated signaling pathway

**Fig 4:**
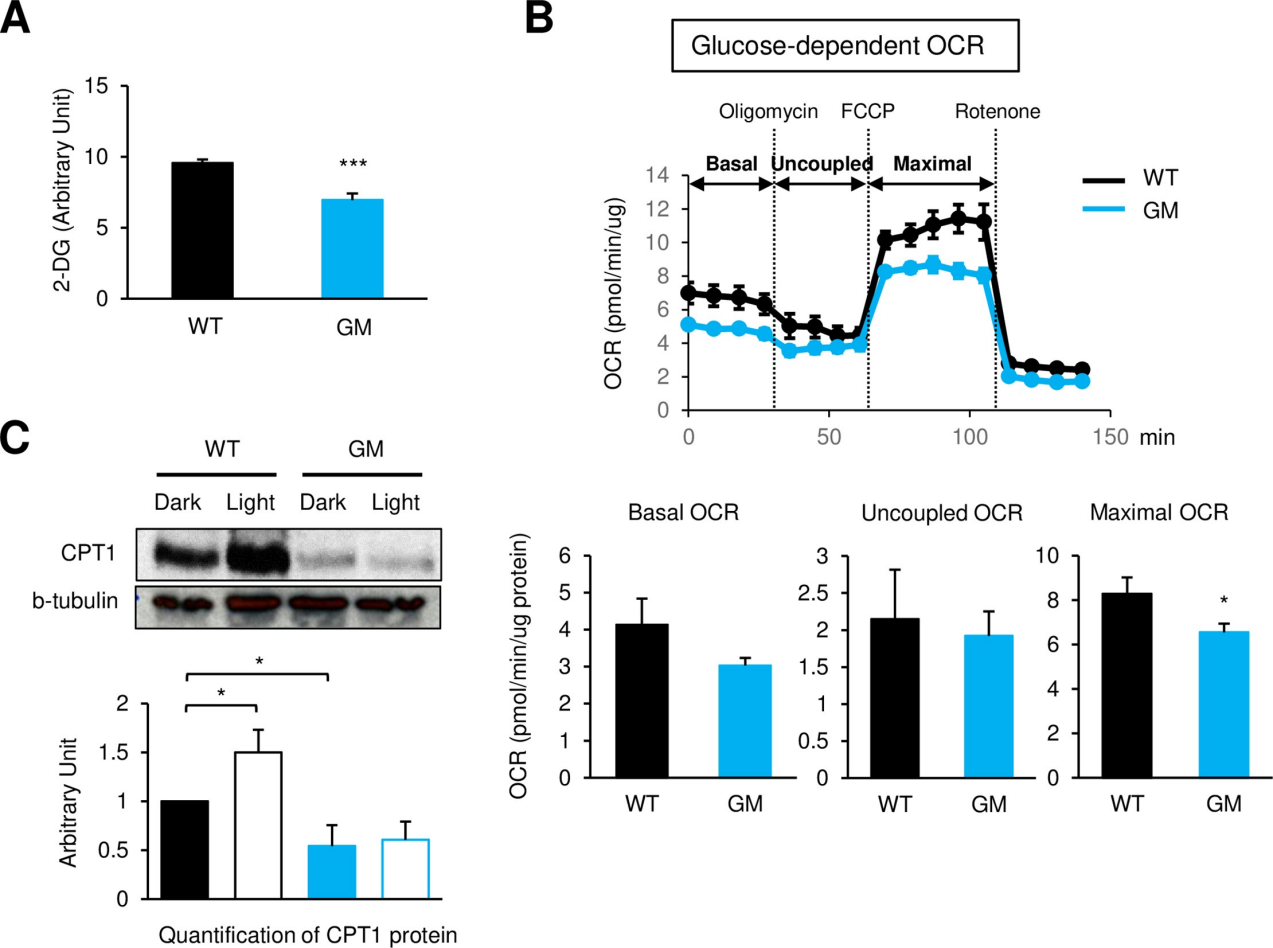
Opn3 regulates fuel intake and usage in brown adipocytes through GPCR signaling. (A) Glucose uptake in differentiated WT and *Opn3*-GM brown adipocytes (*n* = 5–7). The experiment was repeated independently six times. (B) Upper: Glucose-dependent OCR in differentiated WT and *Opn3*-GM brown adipocytes (*n* = 5). Lower: Quantification of OCR. FCCP (*n* = 5). The experiment was repeated independently three times. (C) Upper: Western blot analysis of CPT1 protein in WT and *Opn3*-GM brown adipocytes differentiated in the light or dark condition. Lower: Quantification of CPT1 protein (*n* = 4). This experiment was repeated four times, and similar results were obtained. In experiments of (A) and (B), cells were cultured and differentiated under the normal dark condition in a CO_2_ incubator. The values denote the mean ± SEM, and comparisons were made by Student *t* test. *p < 0.05; ***p < 0.001. The data for this figure can be found in the Dryad repository: https://doi.org/10.5061/dryad.p5hqbzkkv [70]. 2-DG, 2-deoxy-D-glucose; CPT1, carnitine palmitoyltransferase 1; FCCP, carbonyl cyanide 4-(trifluoromethoxy) phenylhydrazone; GPCR, G protein–coupled receptor; OCR, oxygen consumption rate; Opn3, Opsin3; *Opn3*-GM, mutant of *Opn3*’s G protein–binding region; WT, wild-type.

To determine whether the effects of Opn3 in brown adipocytes involve GPCR signaling, we isolated brown preadipocytes from mice that carried a mutation in the G protein–binding region of Opn3 (mutant of *Opn3*’s G protein–binding region [*Opn3-*GM]), meant to disrupt the interaction with its downstream G protein. *Opn3-*GM brown preadipocytes differentiated normally, as evidenced by equal amounts of AP2 protein expression and lipid accumulation in WT and GM cells (S4A and S4B Fig). Similar to what was observed in *Opn3-*KO adipocytes, expression of *Ucp1* mRNA was significantly decreased in *Opn3-*GM brown adipocytes compared with WT cells (S4C Fig). *Opn3-*GM brown adipocytes also displayed a reduction in basal glucose uptake and glucose-dependent mitochondrial respiration compared with WT cells (Fig 4A and 4B). Both basal and light-induced increase of CPT1 protein in WT cells was markedly reduced in *Opn3-*GM cells (Fig 4C). Taken together, these data demonstrate that the effect of light on the fuel metabolism of brown adipocytes is mediated by photoreceptor Opn3 via a GPCR signaling pathway.

### Identification of Opn3-mediated and light-sensitive genes by RNA-sequencing analysis

**Fig 5:**
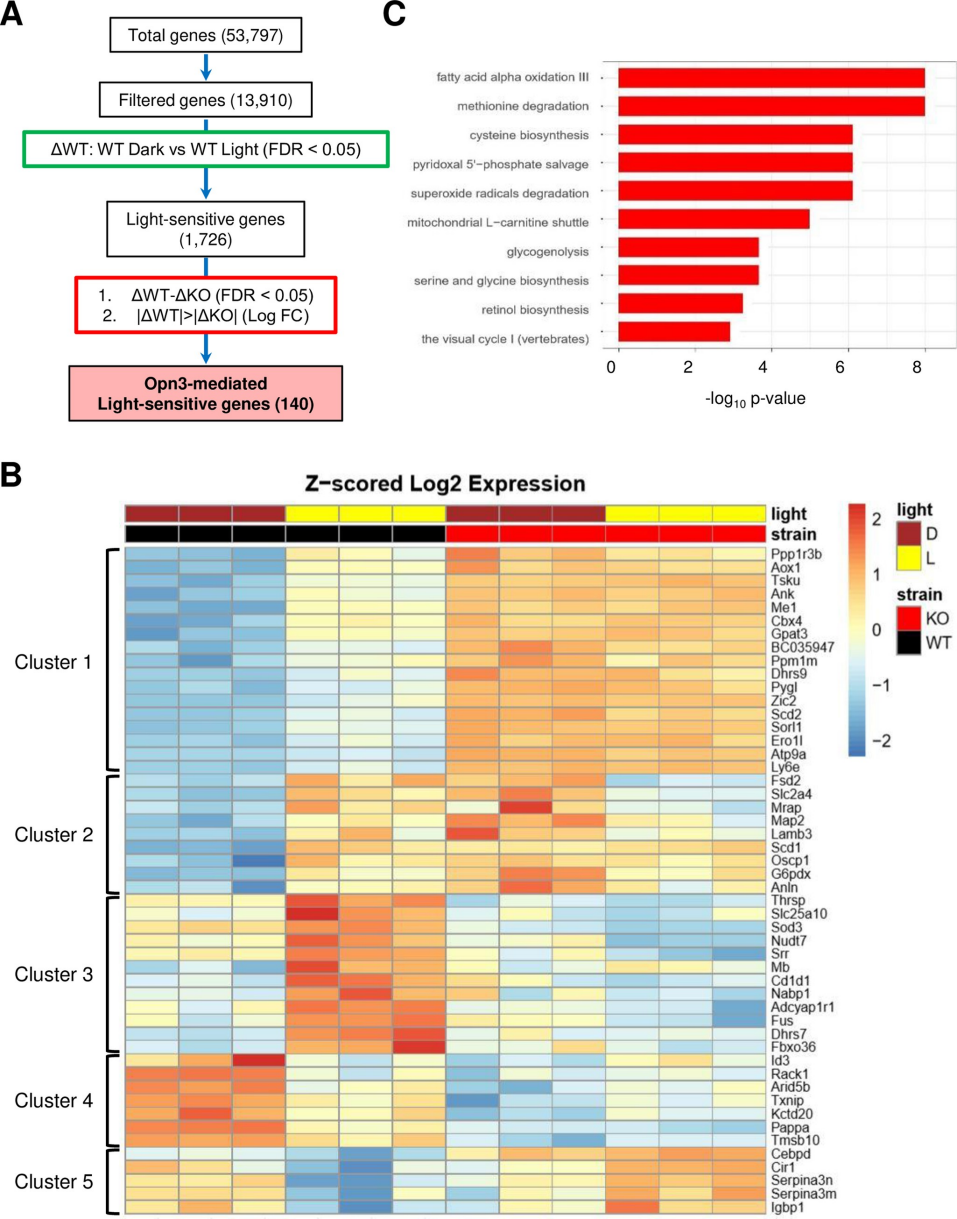
RNA-seq and pathway analysis of Opn3-dependent and light-sensitive genes. (A) Flowchart of analysis for identifying light-sensitive and Opn3-mediated genes. ΔWT and ΔKO are the difference of gene expression between dark and light conditions in WT and *Opn3*-KO cells, respectively. (B) Heat map of the top 50 light-sensitive and Opn3-mediated genes. (C) Bar plot of pathways enriched at FDR < 5% for Opn3-mediated genes. *n* = 3 independent samples per group. The data for this figure can be found in the Dryad repository: https://doi.org/10.5061/dryad.p5hqbzkkv [70]. D, dark; FC, fold change; FDR, false discovery rate; KO, knockout; L, light; Opn3, Opsin3; RNA-seq, RNA-sequencing; WT, wild-type.

To understand the molecular events triggered by light-activated Opn3 in brown adipocytes, we performed RNA-sequencing (RNA-seq) in WT and *Opn3*-KO brown adipocytes that were either exposed to light or kept in the dark, as described above. Among 53,797 transcripts, 1,726 light-sensitive genes were found in WT cells by comparing expression in light conditions to expression in dark conditions (ΔWT) and using a cutoff of less than 5% false discovery rate (FDR). Of these light-sensitive genes, we sought Opn3-mediated genes by identifying transcripts for which light had a greater effect in WT cells than it did in *Opn3*-KO cells. We defined the Opn3-mediated genes as having the following two criteria. First, ΔWT was significantly different from the light-induced change in expression in *Opn3*-KO cells (ΔKO) with a cutoff of less than 5% FDR. Second, the magnitude (absolute value) of ΔWT was greater than the magnitude of ΔKO. Using these criteria, 140 Opn3-mediated light-sensitive genes were identified (Fig 5A).

Next, we sorted the 140 Opn3-mediated genes by significance based on the first criterion described above and plotted the top 50 most significant genes in a heatmap (Fig 5B). We identified five different clusters of genes based on the patterns of gene expression in WT versus *Opn3*-KO cells under dark or light conditions. Among these clusters, cluster 3 represented genes that were highly induced by light in WT cells, and such induction was diminished in *Opn3*-KO cells. Representatives of this cluster included *mitochondrial carrier Solute Carrier Family 25 Member 10* (*Slc25a10*) and *Thyroid Hormone Responsive* (*Thrsp*). Interestingly, *Slc25a10* was previously found to be involved in fatty acid synthesis in adipocytes and insulin sensitivity [29,30]. On the contrary, cluster 4 consisted of genes whose expression was reduced by light in WT cells and maintained low levels of expression in the *Opn3*-KO cells. One of the genes found in this category was *thioredoxin-interacting protein* (*TXNIP*), which is involved in cellular redox signaling [31]. Genes of cluster 1 were induced by light in WT cells, and their expression levels were elevated in *Opn3*-KO cells relative to WT cells, regardless of light or dark condition. Representatives of this cluster included *Tsukushi* (*Tsku*), which is a recently identified hepatokine that can regulate BAT energy expenditure [32], and *Stearoyl-Coenzyme A Desaturase 2* (*Scd2*), which is required for PPARg expression in 3T3-L1 adipose cells [33]. Clusters 2 and 5 included genes that displayed opposite responses to light stimulation between WT and *Opn3*-KO cells.

We also performed pathway analysis for the Opn3-mediated genes and identified enriched pathways using FDR < 5% as the criterion (Fig 5C). Consistent with our in vitro findings, the identified pathways were primarily involved in fatty acid oxidation, amino acid metabolism, and mitochondrial metabolism. For example, fatty acid alpha oxidation III and mitochondrial L-carnitine shuttle pathways were enriched. Carnitine is essential for the transfer of fatty acids to mitochondria for subsequent β-oxidation [34,35], and CPT1 catalyzes carnitine-dependent transport of fatty acids into mitochondria [36]. Thus, these RNA-seq data further supported our functional studies showing a critical role of Opn3 in CPT1–fatty acid mitochondrial respiration. Interestingly, genes of the superoxide radical degradation pathway were also found to be Opn3 regulated (Fig 5C). The mitochondrial electron transport chain utilizes oxygen to produce not only energy but also superoxide [37]. Superoxide is degraded by enzymes such as superoxide dismutase (SOD) to prevent cell damage [38]. It is conceivable that light stimulation through Opn3 increases mitochondrial respiration and, at the same time, triggers an antioxidant defense through the superoxide radical degradation pathway. Together, these data uncover potential molecular mechanisms underlying the cell-autonomous light sensitivity of brown adipocytes and reveal novel light-sensitive genes in nonconventional photoreceptive cells.

### In vivo illumination activates BAT-mediated thermogenesis via Opn3

To investigate whether light can directly stimulate BAT activity via Opn3 in vivo, we established an in vivo optoelectric system for BAT illumination. In this system, the light device is battery-free, and illumination can be controlled and monitored using a wireless closed-loop system [39]. To illuminate with light of a wavelength similar to our in vitro studies, we used white light (combined 465 and 565 nm) as the experimental μLED and red light (650 nm) as the control because mice are naturally insensitive to red light because of their lack of red light–sensitive opsin (long wavelength–sensitive Opsin1) [40,41]. By utilizing the protocol described by Shin and colleagues, 2017 [39], we found that a continuous 12 hours of lighting at 100% duty cycle (20 Hz, 100%, 10 W) led to increased temperature of the device, whereas lighting at 20% duty cycle (20 Hz, 20%, 10 W) did not alter the temperature of the device (S5A Fig), consistent with the previous reference. Thus, we chose the illumination setting at 20% duty cycle (20 Hz, 20%, 10 W), which produced the highest possible light transmission through the tissue with minimal effect on the temperature of the device.

**Fig 6:**
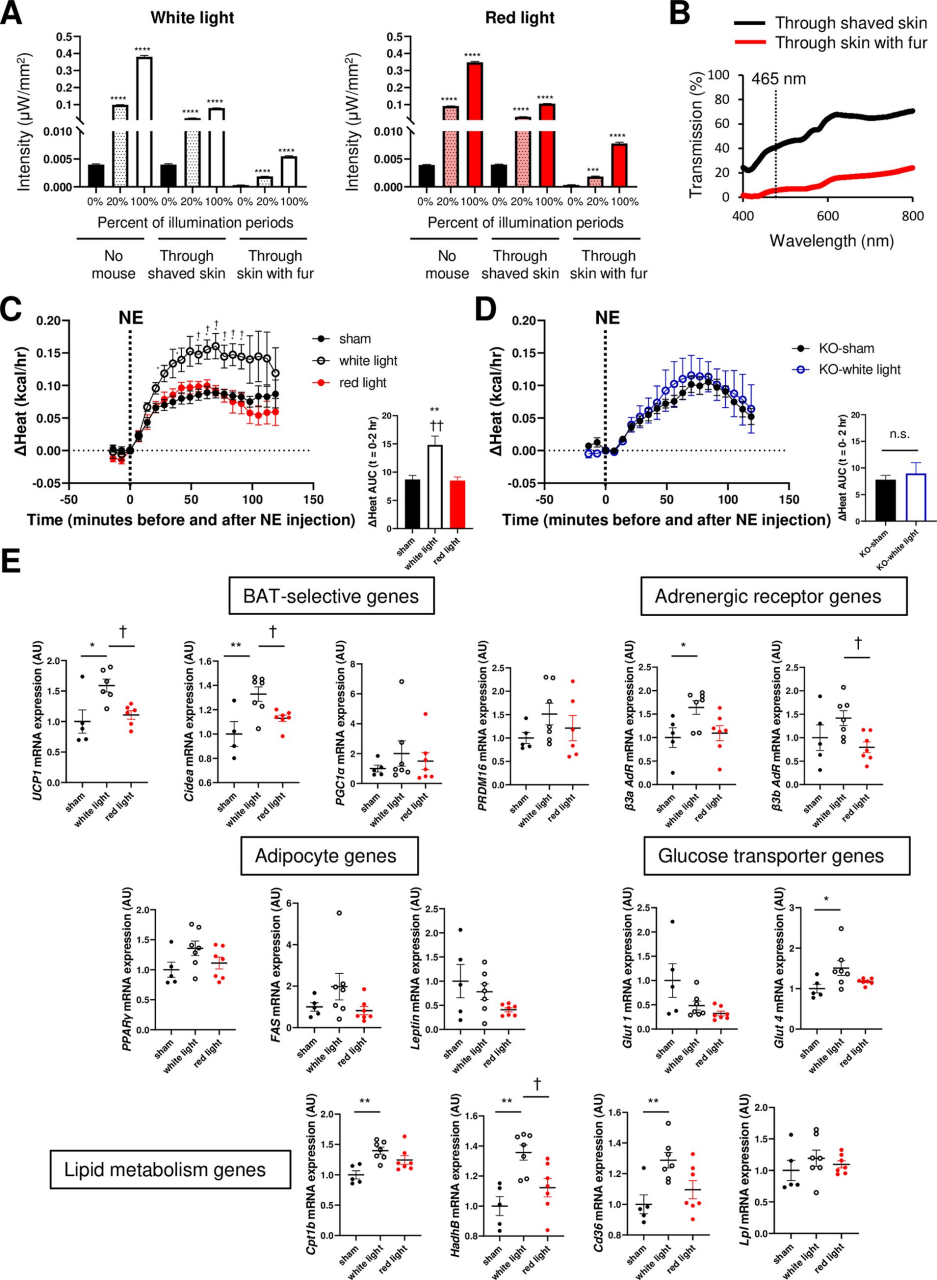
Direct light exposure activates BAT through Opn3 in vivo. (A) The 12-week-old male C57BL/6 mice were anesthetized, and light penetration through the skin on top of BAT was measured in living mice (*n* = 3). Transmitted intensity of white and red light through the shaved neck skin and the skin with fur is shown. Two illumination settings were employed. Pulse frequency: 20 Hz; power: 10 W; percent of illumination periods: 20% or 100%. (B) Transmitted light spectra in the 400- to 800-nm range through shaved skin or skin with fur (*n* = 3, representative data). (C) Left: ΔHeat (energy expenditure) measured by CLAMS for 2 hours in the μLED-implanted and sham-operated mice receiving IP injection of NE (*n* = 6–7). The experiment was performed in 12-week-old male C57BL/6 mice. Right: AUC quantifications of ΔHeat are shown. (D) Left: ΔHeat in littermate *Opn3*-KO mice receiving IP injection of NE (*n* = 3–4). Right: AUC quantifications are shown. (E) Expression levels of genes in BAT of adult male C57BL/6 mice exposed to lighting for 8 days (*n* = 4–7). Data are represented as mean ± SEM. The *p*-values were determined as follows: (A) One-way ANOVA followed by Dunnett’s multiple comparison test. ***p < 0.001, 20% or 100% versus 0% illumination periods at same situations. ([C and D], right, and [E]) One-way ANOVA with Tukey’s multiple comparison test. ([C and D], left) Two-way repeated measures ANOVA followed by Tukey’s multiple comparison test. *p < 0.05, **p < 0.01; *significant difference between light group and sham group. ^†^*p* < 0.05; ^†^significant difference between light wavelength (white and red). The data for this figure can be found in the Dryad repository: https://doi.org/10.5061/dryad.p5hqbzkkv [70]. β3b AdR, β3 adrenaline receptor b; β3a AdR, β3 adrenaline receptor a; AU, arbitrary unit; AUC, area under the curve; BAT, brown adipose tissue; Cidea, Cell-death-inducing DNA fragmentation factor-α-like effector A; CLAMS, Comprehensive Lab Animal Monitoring System; Cpt1b, carnitine palmitoyltransferase 1b; FAS, fatty acid synthase; Glut1, glucose transporter 1; Glut4, glucose transporter 4; HadhB, Hydroxyacyl-CoA dehydrogenase beta subunit; IP, intraperitoneal; KO, knockout; LED, light-emitting diode; Lpl, lipoprotein lipase; NE, norepinephrine; n.s., not significant; Opn3, Opsin3; PGC1α, peroxisome proliferator–activated receptor gamma coactivator-1 alpha; PPARγ, peroxisome proliferator–activated receptor gamma; PRDM16, PR domain containing 16; UCP1, uncoupling protein 1.

To determine whether light is able to penetrate skin and stimulate BAT, we measured light intensity through skin and fur of living mice. We found that both white and red lights can penetrate through skin and fur, and the transmitted intensities increased from illumination at 20%–100% duty cycle (20 Hz, 10 W) (Fig 6A). However, the transmission rate decreased by more than 75% in the presence of fur for both white and red lights. At 465 nm, which is the peak absorbance for vertebrate Opn3 [14], light transmission dropped to 40% through shaved skin, and it became less than 10% through skin with fur (Fig 6B). Thus, although light can penetrate through skin and fur, its immediate ability to activate Opn3 is considerably attenuated. However, under the animal’s natural conditions, there is a prolonged temporal integration of light signal, which may lead to a substantial effect.

To address whether light exposure directly activates BAT function, C57BL/6 mice were divided into three groups and implanted with light devices (white group, white μLED implanted; red group, red μLED implanted; sham group, white μLED implanted and light off). Light devices were implanted on the top of the interscapular BAT (S5D Fig, left and middle). At 7 days after recovery from the surgery, light illumination was activated for 8 days (S5D Fig, right). Importantly, maximum thermogenic capacity, as measured by heat production and VO_2_ in response to NE stimulation, was significantly elevated in the white light group but not in the red light group compared with the sham group (Fig 6C and S5B Fig). Furthermore, these white light–induced effects essentially disappeared in *Opn3*-KO mice (Fig 6D and S5C Fig). At the molecular level, white light increased lipid metabolism genes, including *Cpt1b*, *Hydroxyacyl-CoA dehydrogenase beta subunit* (*HadhB*), and *Cd36* mRNA expression, as well as BAT-selective genes, such as *Ucp1*, *Cell-death-inducing DNA fragmentation factor-α-like effector A* (*Cidea*), *Glucose transporter 4* (*Glut4*), and *β3 adrenaline receptor* gene, compared with sham or red light control groups (Fig 6E). Expression of general adipocyte genes, such as *PPARg*, *Fatty acid synthase* (*FAS*), and *leptin*, was not altered by light exposure compared with sham. These results demonstrate that white light can directly stimulate BAT thermogenic function through an Opn3-dependent mechanism.

## Discussion

In this report, we demonstrate that photoreception through Opn3-GPCR can regulate fuel metabolism and mitochondrial respiration in brown adipocytes. In vivo, we found that direct exposure of BAT to light increased thermogenic capacity in an Opn3-dependent manner. Opn3 was initially identified in murine brain tissue and classified as a GPCR based on its amino acid sequence [12]. Opn3 is classified as a member of the transducin (Gt)-type G protein–coupled Opsin [42,43]. Because Opsins are membrane proteins [43], Opn3 is also predicted to localize on cell membrane and can activate GPCR signaling. Although the absorption spectrum and the biochemical characteristics of mammalian Opn3 have not been reported, other vertebrate Opn3s, such as those from zebrafish and chicken, are capable of activating G proteins in a light-dependent manner when expressed in mammalian cells [44]. *Opn3*-GM cells carry an ERY (Glu-Arg-Tyr)-to-RDR mutation in the G protein–binding motif of Opn3. This highly conserved ERY motif is located at the cytoplasmic end of the third transmembrane domain of class A GPCRs (rhodopsin family) [45,46] and couples receptor conformational states to G protein activation in several GPCRs [47–49], including rhodopsin [50–52]. As such, ERY motif mutants of rhodopsin [50,51], V2 vasopressin receptor [53], and the gonadotropin-releasing hormone receptor [54] have all led to defective signal transduction by affecting the Guanosine diphosphate (GDP)-release step in the respective G protein activation cascades. For example, the interaction between the REY mutant in rhodopsin and its downstream Gt protein decreases by 7,000 times with little or no change in the mutant protein’s expression and retinal morphology [51]. The same may apply to the *Opn3*-GM mutant in question. Unfortunately, there is currently no reliable antibody against Opn3 available for checking its expression level in adipose tissue. In any case, it seems quite likely that the functional defect in *Opn3*-GM brown adipocytes does result from an absence of, or exceedingly weak, GPCR signaling.

Our results reveal that the lack of Opn3 in brown adipose cells leads to impaired lipolysis and decreased mitochondrial activity even in darkness, presumably reflecting some basal constitutive Opn3 activity that exists in adipose tissue without light (as exemplified by rhodopsin; [51]). We employed the RNA-seq approach and identified the top 30 most significantly different genes between WT and *Opn3*-KO cells in the dark (ranked by *p*-value) (S1 Table). These genes are light-insensitive and Opn3-associated genes in the dark. Hence, Opn3 plays a role in brown adipocyte function by regulating both light-sensitive genes and light-insensitive genes.

Increasing energy expenditure by activating BAT is a potential therapeutic approach to treating and preventing obesity and its associated metabolic diseases [55,56]. Cold exposure and the subsequent increase in adrenergic tone are well-known stimulants for the activation of BAT [57], and several metabolic and hormonal signals can also activate brown fat function [3,58]. In this study, we identified light as a novel regulator of BAT, which involves a previously unknown Opn3-GPCR light-sensing mechanism in the regulation of fuel utilization and mitochondrial respiration in brown adipocytes. Furthermore, our in vivo illumination studies provide direct evidence for a light-induced activation of BAT thermogenesis via Opn3. These findings offer both novel insight into the role of light sensing in nonconventional photoreceptive cell types and a potential molecular foundation for further evaluation of light-based therapy.

## Materials and methods

### Ethics statement

All animal procedures were approved by the Institutional Animal Care and Use Committee (IACUC) at Joslin Diabetes Center. All experiments were performed in accordance with the relevant guidelines and regulations described in the IACUC-approved protocol number 09–01.

### Mice and experiments

*Opn3*-KO and *Opn3*-GM mice were generated in Dr. King-Wai Yau’s laboratory at Johns Hopkins University and characterized at the Joslin Diabetes Center. The procedure for making *Opn3*-KO mice was performed as described previously [15], and all experiments using *Opn3*-KO mice were carried out using littermates. The generation of *Opn3*-GM mice is described below. Mice were fed an HFD at 4–5 weeks of age for 13 weeks. Food intake and total fat volume were measured at 17–18 weeks old. The glucose tolerance test (2 g dextrose per kilogram body weight) and the insulin-tolerance test (1 U/kg body weight, Human R, Lilly) were performed in unrestrained conscious mice that were fasted for 6 hours. Blood was collected from the tail at 15, 30, 60, and 120 minutes after injection. Glucose concentrations were determined from blood using Infinity Blood Glucose Meter (US Diagnostics).

### Generation of *Opn3*-GM mutation mice

The *Opn3*-GM mice carry an ERY-to-RDR mutation at the G protein–binding motif of Opn3. These mice were generated by using the CRISPR/Cas9 system. Briefly, the CRISPR Design Tool (http://crispr.mit.edu/) was used for selecting one single-guide RNA (sgRNA) target sequence (5′-CTA TGA ACG TTA TAT CCG TGT GG-3′) close to the desired mutation site in the mouse Opn3 gene. According to (http://crispr.mit.edu/) online protocol, complementary DNA oligos were synthesized, annealed, and cloned into the pX330 vector (addgene) upstream of the *trans*-activating CRISPR RNA (tracrRNA) sequence to generate a chimeric sgRNA construct. PCR-amplified product of this chimeric sequence was used as a template for in vitro transcription with the T7 Quick High Yield RNA Synthesis Kit (New England Biolabs). Purified sgRNA was mixed with Cas9 mRNA (TriLink Biotechnologies) and a 170-bp synthesized oligo (5′-AGG GAC TTA CCC CTA TCC ATA TTC TAC TTT CCT TTC TTG CAG GGT TCG TTT CCA TTA CCA CCC TCA CTG TGC TGG CCT ATC GCG ACA GGA TCC GTG TGG TAC ATG CCA GAG TGA TCA ACT TTT CCT GGG CCT GGA GGG CCA TTA CCT ATA TCT GGC TCT ACT CCT TGG CA-3′, Integrated DNA Technologies) for homology-directed repair. The mixture for sgRNA and Cas9 mRNA was injected into the pronuclei of C57BL/6 embryos at the Transgenic Core Laboratory of Johns Hopkins University School of Medicine. Animals with the targeted mutation were identified by PCR on tail DNA and confirmed by sequencing. A set of primers were used to genotype the mutated allele: Opn3-GM For, 5′-TTG GCA GAA CTT TGG ACA GAG GC-3′; and Opn3-GM Rev, 5′-A TGT GGG CAT CGT TTG GAG AGG-3′. PCR products were directly digested by Nru I (buffer3.1, NEB). WT gave a 650-bp single band, and mutated allele gave a 325-bp single band. To reduce any potential off-target effect, the line has further been crossed at least three times to C57BL/6 mice. Genomic PCR did not reveal any unintended mutations at off-target sites predicted by the CRISPR Design Tool.

### Measurement of light penetration in vivo

Mice were fully anesthetized by pentobarbital (50 mg/kg body weight, IP injection). Prior to surgery, mice were given a dose of analgesia (Banamine, 2.5 mg/kg body weight, s.c. injection) to minimize and prevent postoperative pain and distress. Hair on the back of the mice around the interscapular area was shaved, and then the skin incision was made. Blunt dissection was performed to the adipose tissue. An optical sensor (16 × 10 mm) connected to a power meter (Laser Power Meter LP1, SANWA Electric Instrument) was inserted under the skin. The light device was placed on the skin. The light intensity was measured by using a power meter.

### Measurement of light spectra

Shaved skin and skin with fur-covered BAT were derived from adult C57BL6 WT mice. Transmitted light spectra through skin and fur were measured using multichannel spectrometer (Ocean Optics) in the 400- to 800-nm range with integrated collimating lenses (Ocean Optics). Transmitted spectra were corrected by positive control (spectra without any obstacle) and negative control (transmitted spectra through obstacle), which completely shut off light transmission.

### Measurement of device temperature during lighting

Prior to starting the experiment, a thermal probe [39] was programed to automatically record the temperature every 10 minutes. The probes were placed under the μLED light device. Temperature was recorded after nonlighting or lighting by either white or red light for 12 hours under two different illumination settings (pulse frequency: 20 Hz; power: 10 W; percent of illumination periods: 20% or 100%).

### In vivo illumination experiments

Mice were fully anesthetized by pentobarbital (65 mg/kg body weight, IP injection). Prior to surgery, mice were given a dose of analgesia (Banamine, 2.5 mg/kg body weight, s.c. injection) to minimize and prevent postoperative pain and distress. Hair on the back of the mice around the interscapular area was shaved, and then the skin incision was made right over the BAT. Blunt dissection was performed down to the adipose tissue. A single light device tethered centrally was implanted on the BAT. The device (brain device without indicator, NeuroLux, United States) was sutured to the trapezius muscle on the back of the neck using a monofilament thread (5–0). After the implantation surgery, the open skin was sutured with silk threads (5–0). Mice were given access to food and water and ample bedding for nesting and were monitored regularly for any signs of distress or illness. Mice were allowed 7 days of recovery before turning on the light via the wireless controller box (NeuroLux Optogenetics system, NeuroLux). The light was on for 24 hours per day for 8 days. The light device is battery-free and was controlled and monitored by a wireless closed-loop system. The information for this system, including the light devices and hardware, was obtained from NeuroLux (http://www.neurolux.org/).

### Measuring maximum thermogenic capacity

Mice were fully anesthetized by pentobarbital (65 mg/kg body weight, IP injection). The mice were placed in the Comprehensive Lab Animal Monitoring System (CLAMS, Columbus Instruments) cages for 10 minutes until they were fully asleep, and then CLAMS measurement was started. After the fourth measurement for each mouse, mice were injected with NE (1 mg/kg body weight, s.c. injection) and monitored for an additional 120 minutes. Data for VO_2_, carbon dioxide consumption (VCO_2_), and energy expenditure (Heat) data were normalized to the basal level prior to NE injection (ΔVO_2_, ΔVCO_2_, and ΔHeat) and analyzed among white light, red light, and sham groups.

### Cell isolation and culture

Brown adipocytes and their precursors were isolated from newborn WT *Opn3*-KO and *Opn3*-GM mice by collagenase digestion and were immortalized as described previously [22]. Cells were differentiated into adipocytes for 8 days according to the protocol described in the previous study [59]. Three independent immortalized *Opn3*-KO cell lines were used to measure differentiation capacity, *Ucp1* mRNA expression, and glucose uptake, as shown in S2 Fig. Human brown adipocytes were generated in our laboratory and cultured as previously described [6].

### Light exposure in vitro

The cell culture dish was placed inside the black box that had white LED lights (RTGS Products) attached to the cap of the box. The black box was kept in an incubator, and cells were differentiated for 8 days in this condition. Steady-state photoluminescence spectra of LED light were recorded on a JASCO FP-6500 (Jasco), and the spectra were corrected for detector nonlinearity.

### DNA transfection and isolation of stable Opn3-overexpressing cells

Mouse Opn3 expression vector (EX-Mm02247-M67) and negative control vector (EX-NEG-M67) were purchased from GeneCopoeia. *Opn3*-KO brown preadipocytes were transfected with these plasmids using Xfect transfection reagent (Takara). After transfection, 250 μg/ml hygromycin was added to select hygromycin resistance cells, which stably express Opn3. Cells were cultured with hygromycin-containing medium for 2 weeks to obtain the Opn3-overexpressing cell line.

### Glucose uptake assay

After serum starvation in low-glucose DMEM medium (Gibco) for 4 hours, differentiated brown adipocytes were washed with a HEPES buffer. Glucose transport was determined by the addition of 2-deoxy-[3H] glucose (0.1 mM, 0.5 μCi/ml; PerkinElmer Life and Analytical Science). After 5 minutes of incubation, the reaction was stopped with an ice-cold 0.9% NaCl buffer. Cells were then lysed in 0.1% SDS, and glucose uptake was assessed in 4 ml of scintillant using a Beckman LS6500 scintillation counter (Beckman Coulter). Nonspecific 2-deoxy-[3H] glucose uptake was measured in the presence of cytochalasin B (20 μM) and was subtracted from the total uptake to get the specific glucose uptake. Results were expressed as the mean ± SEM of the indicated number of experiments. The protein content was determined by the Bradford method (Figs 2B, 3B and 4A).

Glucose uptake was measured by the glucose uptake fluorometric assay kit (Sigma) following the manufacturer's protocol. Briefly, after overnight serum starvation in high-glucose DMEM medium (Gibco), differentiated brown adipocytes were starved of glucose in Krebs-Ringer-Phosphate-HEPES buffer for 40 minutes. After the starvation, 2-DG was added and incubated for 20 minutes. Then, cells were lysed, and 2-DG uptake was determined by a coupled enzyme assay in which the phosphorylated 2-DG, 2-DG6P, is oxidized, resulting in the generation of NADPH, which reacts with the probe to generate a fluorometric product. Results were expressed as the mean ± SEM of the indicated number of experiments. The protein content was determined by the Bradford method (Fig s2D and 3E and S2L Fig).

### Lipolysis assay

Cells were grown in 96-well tissue culture plates and differentiated for 8 days. Lipolysis assay was performed using Adipocyte Lipolysis Assay Kit (Zen-Bio), following the manufacturer’s protocol. Briefly, differentiated adipocytes were washed and incubated in lipolysis assay buffer with test compounds such as 5 μM CL-316,243 and 100 μM IBMX at 37°C in an incubator. At 3 hours after incubation, glycerol content in the assay buffer was determined.

### Measurement of cellular OCR

Cells were seeded onto gelatin-coated Seahorse plates and differentiated for 8 days. Cells were serum-starved for 1 hour prior to the beginning of the experiment. OCR was monitored in a Seahorse Bioanalyzer XF24 instrument (Agilent) using oligomycin (1 μM), FCCP (1.2 μM), and rotenone (1 μM), allowing four measurements after each injection. Additionally, we utilized 25 mM glucose or 2.5 mM glucose plus 0.5 mM carnitine and 0.175 mM palmitate-BSA (in the running medium) as fuel, and 100 μM etomoxir (Sigma) was used to block CPT1 activity. For normalization of respiration to protein content, cells were lysed in RIPA buffer, and protein concentrations were measured using the Pierce BCA kit (Life Technologies).

### Western blotting

Cells were lysed in a RIPA buffer, and protein concentrations were determined by using the Pierce BCA kit (Life Technologies) according to the manufacturer’s instructions. Equal protein concentrations (determined by Bradford assay) were loaded onto an SDS-PAGE gel. Proteins were then transferred to a PVDF membrane and probed with antibodies for Glut1 (Abcam, ab115730), CPT1-M (Alpha Diagnostic, CPT1M11-A), UCP1 (Abcam, ab10983), PPARγ (Santa Cruz Biotechnology, sc-7273), HSL (#18381), Phospho-HSL (Ser563: #4139, Ser565: #4137, Ser660: #4126), ATGL (#2439), FABP4 (AP2: #3544), GAPDH (#2118), and b-tubulin (#2146) (all from Cell Signaling), followed by incubation with appropriate secondary antibodies and visualization with enhanced chemiluminescence substrate.

### RNA-seq

Total RNA was extracted from cells using the spin column kit (Zymo Research). The integrity and purity of total RNA were assessed using an Agilent 2100 Bioanalyzer (Agilent Technologies). The Illumina TruSeq Stranded mRNA protocol was used for the preparation of RNA-seq libraries and sequenced on a HiSeq 2500 machine (Illumina) as paired-end, 100-bp reads.

### RNA-seq analysis

Paired-end sequencing reads from HiSeq 2500 were subjected to adaptor trimming using cutadapt version 1.1 (https://cutadapt.readthedocs.io/en/stable/) and were quality trimmed using Trimmomatic version 0.32 [60]. We next used TopHat (version 2.0.14 [61]) with the default parameters to map the reference genome (GRCm38, mm10). Counts were calculated using Cufflinks (version 2.2.1 [62]) with a transcriptome reference (Ensembl Mouse Transcript). Of 53,797 transcripts, we filtered out those that do not have at least 1 count per million (CPM) in at least three samples, leaving 13,910 transcripts. We normalized the counts with the TMM method [63]. We then transformed count data to log_2_-counts per million (logCPM), estimated the mean–variance relationship, and used this to compute observation-level weights, which allows for linear regression modeling with the Limma package [64,65]. We then tested group comparisons in each gene with Limma’s empirical Bayes linear regression models and accounted for multiple testing with the Benjamini-Hochberg FDR. We tested enrichment of HumanCyc metabolic pathways [66] obtained from Harmonizome [67] in our list of Opn3-mediated genes with Limma’s CAMERA preranked method [68], which accounts for correlations between genes.

### Quantitative RT-PCR

Total RNA was extracted from cells with Trizol and purified using a spin column kit (Zymo Research). RNA (500–1,000 ng) was reverse transcribed with a high-capacity complementary DNA (cDNA) reverse transcription kit (Applied Biosystems). qRT-PCR assays were run and quantified in the ABI Prism 7900 sequence-detection system using SYBR green PCR Master Mix (Roche). Relative mRNA expression was determined by the ΔCt method, and the values were normalized to the expression of Acidic ribosomal phosphoprotein P0 (ARBP). The sequences of primers used in this study are provided in S2 Table.

### Quantification of mitochondrial DNA content by qPCR

DNA was isolated from WT and *Opn3*-KO adipocytes at day 8 of differentiation. ND1 and ND6 primers were used to evaluate mitochondrial DNA, and GAPDH primers designed to target an intronic region of the gene were used to measure nuclear DNA. qPCR was performed with 50 ng of DNA, and ND1 and ND6 expression level was normalized to expression of GAPDH. The primer sequences are noted in S2 Table.

### Oil Red O staining

To stain the lipid droplets inside of cells, Oil Red O staining was performed. Cells were washed twice with PBS and fixed with 10% buffered formalin overnight at 4°C. Cells were then stained for 2–4 hours at room temperature with a filtered Oil Red O solution (0.5% Oil Red O in isopropyl alcohol), washed twice with distilled water, and then visualized.

### Fatty acid uptake assay

Fatty acid uptake was determined by measuring [14C] palmitic acid uptake as previously described [69].

### Cytochrome oxidase assay

Mitochondrion was isolated from differentiated brown adipocytes by mitochondrial isolation kit (Abcam). We measured mitochondrial complex IV activity using a cytochrome c oxidase assay kit (SIGMA) following the manufacturer’s protocol. Briefly, in a 1-ml cuvette, we added reduced cytochrome c and cytochrome c oxidase assay buffer to 3 μg of mitochondrial protein and recorded the decrease in absorbance at 550 nm over 10 minutes in spectrometer (Denovix). The decrease in absorbance is proportional to an increase in oxidized cytochrome c. We presented cytochrome oxidase activity as (Units/mg) = [(Absorbance1 − Absorbance2)/(t1 –t2)]/(21.84 × volume of samples). One unit will oxidize one μ mole of reduced cytochrome c per minute.

### Statistical analysis

All statistics were calculated using Microsoft Excel and GraphPad Prism. All data were presented as mean ± SEM, and comparisons were made by Student *t* test, one-way ANOVA, and two-way repeated measures ANOVA as appropriate. Details are described in each figure legend. Results were considered significant if *p < 0.05, **p < 0.01, or ***p < 0.001. All experiments were performed on at least two to three independent experiments, except the RNA-seq study. The experiments were not randomized, and the sample size was not predetermined.

## Supporting information

S1 Fig Opsins expression and metabolic analysis in Opn3-KO mice. (A) Littermate mice were fed with NCD, and body weight was measured every week (*n* = 4–6) (B) Left: GTT was performed after 12 weeks of HFD. Mice were fasted for 6 hours, followed by an IP injection of dextrose. Blood glucose levels were determined at the indicated times after injection. Right: AUC for the GTT was calculated for respective time interval (*n* = 8–13). (C) ΔVO_2_ measured by the CLAMS for 35 minutes in *Opn3*-KO and WT mice fed with NCD receiving IP injection of NE under the NCD condition (*n* = 6). Right: AUC quantifications of ΔVO_2_ is shown. (D) *Opn3* and *Opsin1MW 2*, *4*, and *5* mRNA expression in brown adipose tissue dissected from adult littermate WT and *Opn3*-KO mice (10- to 11-week-old, *n* = 6−7). Data are represented as mean ± SEM. The *p*-values were determined by two-way repeated measures ANOVA followed by Bonferroni’s test ([C], left) and unpaired *t* test ([C], right, and [D]). **p < 0.01, ****p < 0.0001. The data for this figure can be found in the Dryad repository: https://doi.org/10.5061/dryad.p5hqbzkkv [70]. AUC, area under the curve; CLAMS, Comprehensive Lab Animal Monitoring System; GTT, glucose tolerance test; HFD, high-fat diet; IP, intraperitoneal; KO, knockout; *MW*, medium wavelength; NCD, normal chow diet; NE, norepinephrine; Opn3, Opsin3; VO_2_, oxygen consumption; WT, wild-type.
(TIF)

S2 Fig Gene expression and metabolic analysis in WT and Opn3-KO brown adipose cells. (A) *Opsins* mRNA expression in WT brown adipocytes at day 0 (*n* = 3). The experiment was performed in three technical replicates. (B) *Opn3* mRNA expression in WT brown adipocytes during the course of differentiation (day 0–8) (*n* = 3). (C) *Opsins* mRNA expression in human brown preadipocytes (*n* = 3). (D) *AP2* mRNA expression (*n* = 3). (E, F) Western blot analysis of AP2 and PPARg protein level in WT and *Opn3*-KO brown adipocytes and quantification of AP2 (*n* = 4) and PPARg (*n* = 3) protein. The experiment was repeated independently three times. (G) Upper: Lipid droplets in WT and *Opn3*-KO brown adipocytes at day 8 of differentiation were stained by Oil red O staining (see Materials and methods). Lower: Quantification of Oil red O staining (*n* = 6). The experiment was conducted in three independent biologically independent experiments. (H) mRNA (left, *n* = 3) and protein (right, quantification was *n* = 5) of *Ucp1*, a specific marker for brown adipose tissue, expression in WT and KO brown adipocytes at day 0 and day 8 of differentiation. These experiments were performed in three biological independent experiments. (I) *AP2* mRNA expression was measured in two other immortalized *Opn3*-KO brown adipose cell lines at day 8 of differentiation (*n* = 3). The experiment was performed in three biological independent experiments. (J) Oil red O staining was performed with two other immortalized *Opn3*-KO cell lines at day 8 of differentiation, and the staining was quantified (*n* = 6). (K) *Ucp1* mRNA expression was measured in two other immortalized *Opn3*-KO brown adipose cell lines at day 8 of differentiation (*n* = 3). The experiment was performed in three biological independent experiments. (L) Glucose uptake of two other immortalized *Opn3*-KO brown adipose cell lines. (*n* = 7–8). The experiment was performed in three biological independent experiments. (M) *Opn3* and *Ucp1* mRNA expression was measured in WT cells, *Opn3*-KO, and *Opn3*-KO + Opn3-OE cells at day 8 of differentiation (*n* = 3). The experiment was performed in three biological independent experiments. (N) Left: Western blot analysis of HSL and ATGL protein levels in WT and *Opn3*-KO cells. This experiment was repeated three times with similar results. Right: Quantification of ATGL is shown (*n* = 3). (O) Fatty acid uptake of differentiated WT and *Opn3*-KO brown adipocytes (*n* = 10). The experiment was repeated independently two times. (P) mtDNA content was determined by qPCR with genomic DNA. mtDNA-specific ND1 and ND6 normalized to nuclear specific gene GAPDH (*n* = 3). This experiment was repeated three times with similar results. (Q) Left: Measurement of the decrease in absorbance at 550 nm of reduced cytochrome c caused by its oxidation by cytochrome c oxidase contained in mitochondrial protein of differentiated WT and *Opn3*-KO cells (*n* = 3). Absorbance decreases indicate an increase in cytochrome c oxidase activity. Right: Cytochrome c oxidase activity defined by the rate of change in the linear change (*n* = 6, see Materials and methods). The experiment was repeated independently three times. In all of the above experiments, cells were cultured and differentiated under the normal dark condition in a CO_2_ incubator. The values denote the mean ± SEM, and comparisons were made by Student *t* test ([A–L] and [N–Q]) or one-way ANOVA followed by a Tukey’s post hoc test (M). *p < 0.05; **p < 0.01; ***p < 0.001. The data for this figure can be found in the Dryad repository: https://doi.org/10.5061/dryad.p5hqbzkkv [70]. *AP2*, *adipocyte protein 2*; ATGL, adipose tissue triglyceride lipase; GAPDH, Glyceraldehyde-3-phosphate dehydrogenase; HSL, hormone-sensitive lipase; KO, knockout; mtDNA, mitochondrial DNA; ND1, NADH dehydrogenase subunit 1; ND6, NADH dehydrogenase subunit 6; Opn3, Opsin3; Opn3-OE, overexpression of *Opn3*; PPARg, peroxisome proliferator–activated receptor g; qPCR, quantitative polymerase chain reaction; *Ucp1*, *uncoupling protein-1*; WT, wild-type.
(TIF)

S3 Fig Light-induced metabolic changes in WT and Opn3-KO brown adipocytes. (A) Spectrum of white LED light. (B) Left: Cells were cultured with or without light stimulation, and the temperature of medium was monitored every 1 hour for 24 hours. Right: An average temperature of 24 hours. The experiment was repeated independently two times. (C) *Opn3* mRNA expression was measured in WT and *Opn3*-KO brown adipocytes differentiated in light or dark conditions (*n* = 3). (D) Quantification of OCR shown in Fig 3C (*n* = 10–11). (E) Lipolysis assay of differentiated WT and *Opn3*-KO brown adipose cells stimulated with vehicle, CL-316,243 (5 μM), or IBMX (100 μM) (*n* = 3). The experiment was repeated independently two times. (F) Western blot analysis of ATGL protein level in WT and *Opn3*-KO brown adipocytes differentiated in light or dark conditions and the quantification of band is shown (*n* = 3). This experiment was repeated three times with similar results. (G) mtDNA content was determined by qPCR with genomic DNA. mtDNA-specific ND1 and ND6 normalized to nuclear specific gene GAPDH (*n* = 3). This experiment was repeated three times with similar results. (H) Cytochrome c oxidase activity (see Materials and methods) of differentiated WT and *Opn3*-KO brown adipocytes with or without light stimulation (*n* = 3). The experiment was repeated independently three times. (I) Quantification of OCR shown in Fig 3D (*n* = 7). (J) The cells were collected at indicated time points after dexamethasone shock, and clock gene expression levels were analyzed by qPCR (*n* = 3). The experiment was performed in three independent technical replicates. The values denote the mean ± SEM, and comparisons were made by Student *t* test. *p < 0.05; **p < 0.01; ***p < 0.001. The data for this figure can be found in the Dryad repository: https://doi.org/10.5061/dryad.p5hqbzkkv [70]. ATGL, adipose tissue triglyceride lipase; GAPDH, Glyceraldehyde-3-phosphate dehydrogenase; IBMX, 3-isobutyl-1-methylxanthine; KO, knockout; LED, light-emitting diode; mtDNA, mitochondrial DNA; ND1, NADH dehydrogenase subunit 1; ND6, NADH dehydrogenase subunit 6; OCR, oxygen consumption rate; Opn3, Opsin3; qPCR, quantitative polymerase chain reaction; WT, wild-type.
(TIF)

S4 Fig Gene expression analysis in Opn3-GM brown adipose cells. (A) AP2 protein expression and quantification at day 8 (*n* = 4). The experiment was repeated independently three times. (B) Upper: Oil red O staining in WT and *Opn3*-GM brown adipocytes at day 8 of differentiation. Lower: Quantification of cells stained with Oil red O (*n* = 4). The experiment was repeated independently two times. (C) Left: mRNA expression of *Ucp1*, a specific marker for brown adipose tissue, and expression in WT and *Opn3*-GM brown adipocytes at day 0 and day 8 of differentiation (*n* = 3). Right: Ucp1 protein expression and quantification at day 8 of differentiation (*n* = 4). These experiments were repeated independently three times. In all of the above experiments, cells were cultured and differentiated under the normal dark condition in a CO_2_ incubator. The values denote mean ± SEM, and comparisons were made by Student *t* test. **p < 0.01. The data for this figure can be found in the Dryad repository: https://doi.org/10.5061/dryad.p5hqbzkkv [70]. AP2, adipocyte protein 2; Opn3, Opsin3; *Opn3*-GM, mutant of *Opn3*’s G protein–binding region; *Ucp1*, *uncoupling protein-1*; WT, wild-type.
(TIF)

S5 Fig Direct light stimulation of BAT via Opn3 up-regulated BAT activity in vivo. (A) Left: Device temperature during white lighting, red lighting, or nonlighting was measured every 10 minutes for 12 hours under two different illumination setting (pulse frequency: 20 Hz; power: 10 W; percent of illumination periods: 20% or 100%, *n* = 3–4). Right: Average device temperature for 12 hours (*n* = 3–4). (B) Left: ΔVO_2_ measured by the CLAMS for 2 hours in the μLED implanted and sham-operated mice receiving IP injection of norepinephrine (*n* = 6–7). The experiment was performed with adult male C57BL/6 mice. Right: AUC quantifications of ΔVO_2_ are shown. (C) Left: ΔVO_2_ measuring in littermate *Opn3*-KO mice receiving IP injection of norepinephrine (*n =* 3–4). Right: AUC quantifications are shown. (D) Pictures of surgery for in vivo illumination. Left and middle: Light device was placed on the top of the interscapular BAT (surrounded by yellow line). Right: Light was activated after the surgical recovery. Arrows indicate lighting. Data are represented as mean ± SEM. The *p*-values were determined as follows: (A) ordinary one-way ANOVA followed by Tukey’s multiple comparison test. **p < 0.01, ***p < 0.001; *significant difference between lighting and nonlighting. ([B] and [C], left) Two-way repeated measures ANOVA followed by Tukey’s multiple comparison test. ([B] and [C], right) Ordinary one-way ANOVA with Tukey’s multiple comparison test. *p < 0.05, **p < 0.01; *significant difference between light group and sham group. ^†^*p <* 0.05, ^††^*p <* 0.01; ^†^significant difference between light wavelength (white and red). The data for this figure can be found in the Dryad repository: https://doi.org/10.5061/dryad.p5hqbzkkv [70]. AUC, area under the curve; BAT, brown adipose tissue; CLAMS, Comprehensive Lab Animal Monitoring System; IP, intraperitoneal; KO, knockout; LED, light-emitting diode; Opn3, Opsin3; VO_2_, oxygen consumption.
(TIF)

S6 Fig Plates of Oil red O staining shown in this manuscript. (TIF)

S1 Table Top 30 most significant genes from comparing WT versus Opn3-KO cells in the dark. KO, knockout; Opn3, Opsin3; WT, wild-type.
(TIF)

S2 Table Primer sequences. (TIF)

S1 Raw Images Original images supporting western blot results reported in Figs 2C, 2F, 3F, 4C and S2E, S2F, S2H, S2N, S3F, S4A and S4C. The data can be found in the Dryad repository: https://doi.org/10.5061/dryad.p5hqbzkkv [70].
(PDF)

## Abbreviations

β3b AdR: β3 adrenaline receptor b
β3a AdR: β3 adrenaline receptor a
AU: arbitrary unit
AP2: adipocyte protein 2
ATGL: adipose tissue triglyceride lipase
AUC: area under the curve
BAT: brown adipose tissue
Cidea: *Cell-death-inducing DNA fragmentation factor-α-like effector A*
CPT1: carnitine palmitoyltransferase 1
Cry1: *cryptochrome-1*
Cry2: *cryptochrome-2*
DEXA: dual-energy X-ray absorptiometry
FAS: *Fatty acid synthase*
FC: fold change
FCCP: carbonyl cyanide 4-(trifluoromethoxy) phenylhydrazone
FDR: false discovery rate
GDP: guanosine diphosphate
Glut1: glucose transporter 1
Glut4: *glucose transporter 4*
GPCR: G protein–coupled receptor
Gt: transducin
HadhB: *Hydroxyacyl-CoA dehydrogenase beta subunit*
HFD: high-fat diet
HSL: hormone-sensitive lipase
IBMX: 3-isobutyl-1-methylxanthine
KO: knockout
LED: light-emitting diode
Lpl: lipoprotein lipase
NCD: normal chow diet
ND1: NADH dehydrogenase subunit 1
ND6: NADH dehydrogenase subunit 6
NE: norepinephrine
OCR: oxygen consumption rate
Opn3: Opsin3
Opn3-GM: mutant of Opn3’s G protein–binding region
Per1: *Period-1*
Per2: *Period-2*
Per3: *Period-3*
PGC1α: peroxisome proliferator–activated receptor gamma coactivator-1 alpha
PPARg: *peroxisome proliferator–activated receptor g*
PRDM16: PR domain containing 16
RNA-seq: RNA-sequencing
Scd2: *Stearoyl-Coenzyme A Desaturase 2*
Slc25a10: *Solute Carrier Family 25 Member 10*
SOD: superoxide dismutase
Thrsp: *Thyroid Hormone Responsive*
Tsku: *Tsukushi*
TXNIP: *thioredoxin-interacting protein*
UCP1: uncoupling protein-1
VO_2_: oxygen consumption
WAT: white adipose tissue
WT: wild-type
