# Supplementary figures and images for: Cell-autonomous light sensitivity via Opsin3 regulates fuel utilization in brown adipocytes
Source: PLoS Biol. 2020 Feb 10;18(2):e3000630. doi: 10.1371/journal.pbio.3000630 (PMC7034924; doi:10.1371/journal.pbio.3000630)

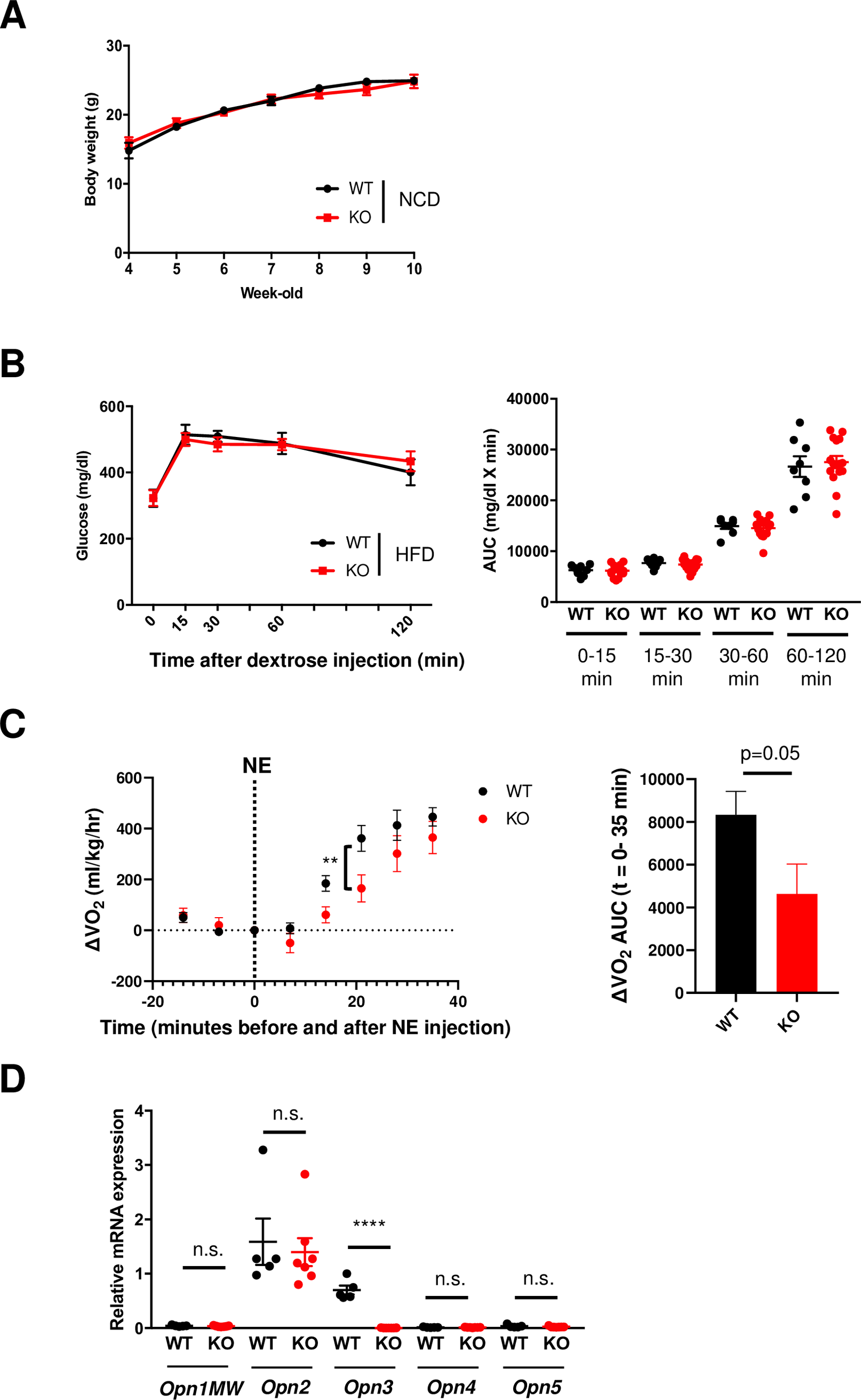

Supplement: S1 Fig — (A) Littermate mice were fed with NCD, and body weight was measured every week (n = 4–6) (B) Left: GTT was performed after 12 weeks of HFD. Mice were fasted for 6 hours, followed by an IP injection of dextrose. Blood glucose levels were determined at the indicated times after injection. Right: AUC for the GTT was calculated for respective time interval (n = 8–13). (C) ΔVO2 measured by the CLAMS for 35 minutes in Opn3-KO and WT mice fed with NCD receiving IP injection of NE under the NCD condition (n = 6). Right: AUC quantifications of ΔVO2 is shown. (D) Opn3 and Opsin1MW 2, 4, and 5 mRNA expression in brown adipose tissue dissected from adult littermate WT and Opn3-KO mice (10- to 11-week-old, n = 6−7). Data are represented as mean ± SEM. The p-values were determined by two-way repeated measures ANOVA followed by Bonferroni’s test ([C], left) and unpaired t test ([C], right, and [D]). **p < 0.01, ****p < 0.0001. The data for this figure can be found in the Dryad repository: https://doi.org/10.5061/dryad.p5hqbzkkv [70]. AUC, area under the curve; CLAMS, Comprehensive Lab Animal Monitoring System; GTT, glucose tolerance test; HFD, high-fat diet; IP, intraperitoneal; KO, knockout; MW, medium wavelength; NCD, normal chow diet; NE, norepinephrine; Opn3, Opsin3; VO2, oxygen consumption; WT, wild-type. (TIF) [file pbio.3000630.s001.tif]

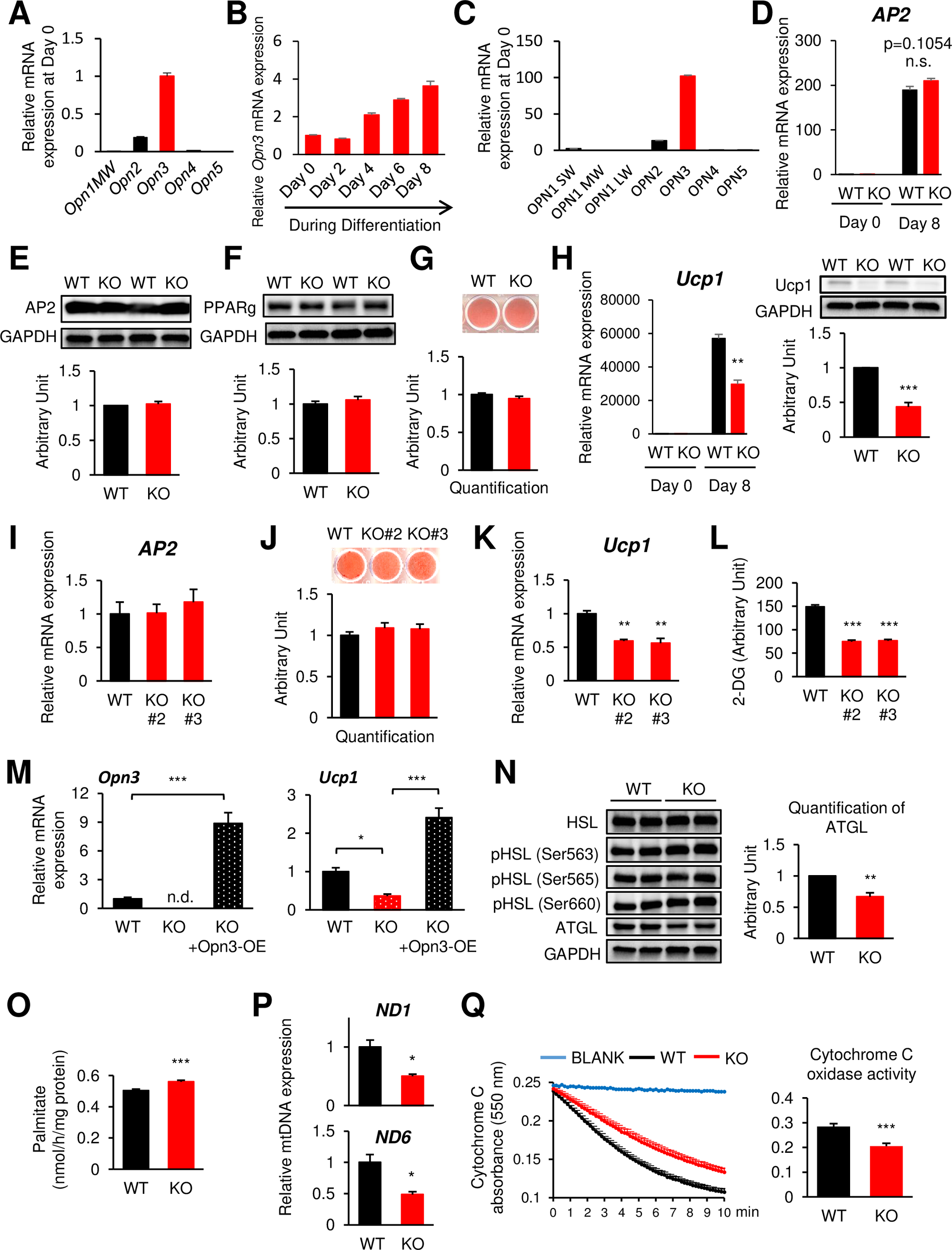

Supplement: S2 Fig — (A) Opsins mRNA expression in WT brown adipocytes at day 0 (n = 3). The experiment was performed in three technical replicates. (B) Opn3 mRNA expression in WT brown adipocytes during the course of differentiation (day 0–8) (n = 3). (C) Opsins mRNA expression in human brown preadipocytes (n = 3). (D) AP2 mRNA expression (n = 3). (E, F) Western blot analysis of AP2 and PPARg protein level in WT and Opn3-KO brown adipocytes and quantification of AP2 (n = 4) and PPARg (n = 3) protein. The experiment was repeated independently three times. (G) Upper: Lipid droplets in WT and Opn3-KO brown adipocytes at day 8 of differentiation were stained by Oil red O staining (see Materials and methods). Lower: Quantification of Oil red O staining (n = 6). The experiment was conducted in three independent biologically independent experiments. (H) mRNA (left, n = 3) and protein (right, quantification was n = 5) of Ucp1, a specific marker for brown adipose tissue, expression in WT and KO brown adipocytes at day 0 and day 8 of differentiation. These experiments were performed in three biological independent experiments. (I) AP2 mRNA expression was measured in two other immortalized Opn3-KO brown adipose cell lines at day 8 of differentiation (n = 3). The experiment was performed in three biological independent experiments. (J) Oil red O staining was performed with two other immortalized Opn3-KO cell lines at day 8 of differentiation, and the staining was quantified (n = 6). (K) Ucp1 mRNA expression was measured in two other immortalized Opn3-KO brown adipose cell lines at day 8 of differentiation (n = 3). The experiment was performed in three biological independent experiments. (L) Glucose uptake of two other immortalized Opn3-KO brown adipose cell lines. (n = 7–8). The experiment was performed in three biological independent experiments. (M) Opn3 and Ucp1 mRNA expression was measured in WT cells, Opn3-KO, and Opn3-KO + Opn3-OE cells at day 8 of differentiation (n = 3). The experiment was [file pbio.3000630.s002.tif]

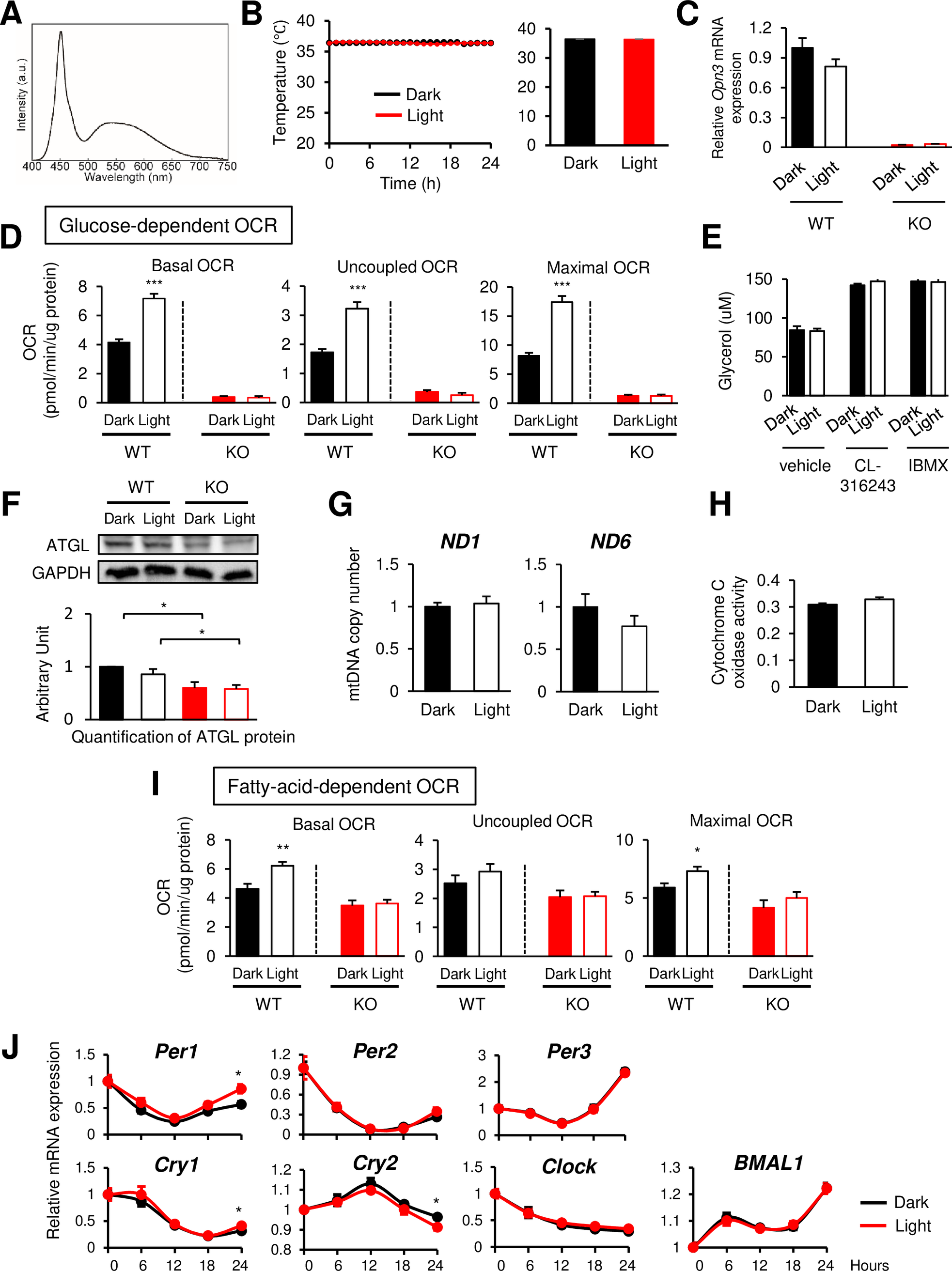

Supplement: S3 Fig — (A) Spectrum of white LED light. (B) Left: Cells were cultured with or without light stimulation, and the temperature of medium was monitored every 1 hour for 24 hours. Right: An average temperature of 24 hours. The experiment was repeated independently two times. (C) Opn3 mRNA expression was measured in WT and Opn3-KO brown adipocytes differentiated in light or dark conditions (n = 3). (D) Quantification of OCR shown in Fig 3C (n = 10–11). (E) Lipolysis assay of differentiated WT and Opn3-KO brown adipose cells stimulated with vehicle, CL-316,243 (5 μM), or IBMX (100 μM) (n = 3). The experiment was repeated independently two times. (F) Western blot analysis of ATGL protein level in WT and Opn3-KO brown adipocytes differentiated in light or dark conditions and the quantification of band is shown (n = 3). This experiment was repeated three times with similar results. (G) mtDNA content was determined by qPCR with genomic DNA. mtDNA-specific ND1 and ND6 normalized to nuclear specific gene GAPDH (n = 3). This experiment was repeated three times with similar results. (H) Cytochrome c oxidase activity (see Materials and methods) of differentiated WT and Opn3-KO brown adipocytes with or without light stimulation (n = 3). The experiment was repeated independently three times. (I) Quantification of OCR shown in Fig 3D (n = 7). (J) The cells were collected at indicated time points after dexamethasone shock, and clock gene expression levels were analyzed by qPCR (n = 3). The experiment was performed in three independent technical replicates. The values denote the mean ± SEM, and comparisons were made by Student t test. *p < 0.05; **p < 0.01; ***p < 0.001. The data for this figure can be found in the Dryad repository: https://doi.org/10.5061/dryad.p5hqbzkkv [70]. ATGL, adipose tissue triglyceride lipase; GAPDH, Glyceraldehyde-3-phosphate dehydrogenase; IBMX, 3-isobutyl-1-methylxanthine; KO, knockout; LED, light-emitting diode; mtDNA, mitochondrial DNA; ND1, NADH dehydrogenase s [file pbio.3000630.s003.tif]

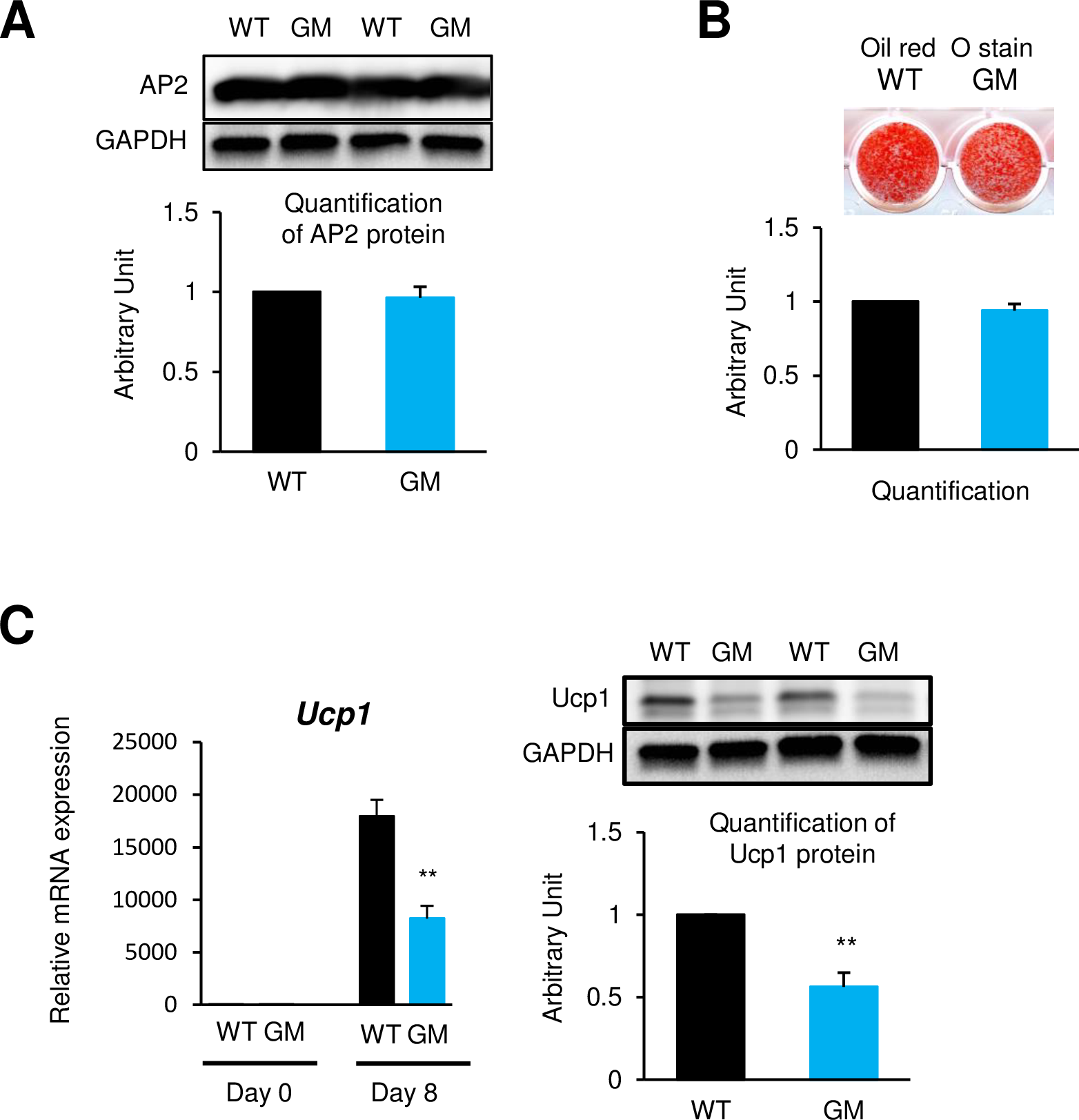

Supplement: S4 Fig — (A) AP2 protein expression and quantification at day 8 (n = 4). The experiment was repeated independently three times. (B) Upper: Oil red O staining in WT and Opn3-GM brown adipocytes at day 8 of differentiation. Lower: Quantification of cells stained with Oil red O (n = 4). The experiment was repeated independently two times. (C) Left: mRNA expression of Ucp1, a specific marker for brown adipose tissue, and expression in WT and Opn3-GM brown adipocytes at day 0 and day 8 of differentiation (n = 3). Right: Ucp1 protein expression and quantification at day 8 of differentiation (n = 4). These experiments were repeated independently three times. In all of the above experiments, cells were cultured and differentiated under the normal dark condition in a CO2 incubator. The values denote mean ± SEM, and comparisons were made by Student t test. **p < 0.01. The data for this figure can be found in the Dryad repository: https://doi.org/10.5061/dryad.p5hqbzkkv [70]. AP2, adipocyte protein 2; Opn3, Opsin3; Opn3-GM, mutant of Opn3’s G protein–binding region; Ucp1, uncoupling protein-1; WT, wild-type. (TIF) [file pbio.3000630.s004.tif]

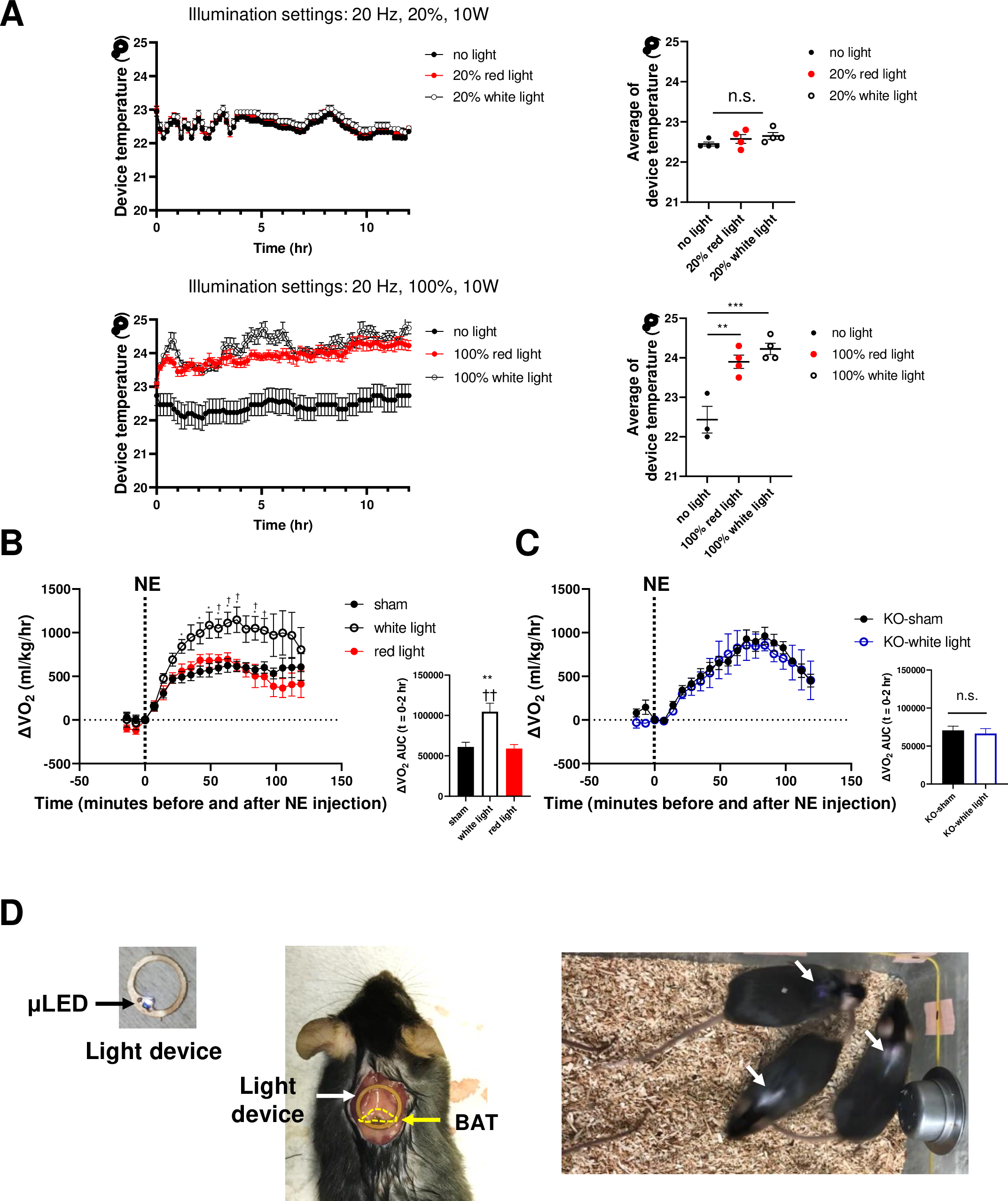

Supplement: S5 Fig — (A) Left: Device temperature during white lighting, red lighting, or nonlighting was measured every 10 minutes for 12 hours under two different illumination setting (pulse frequency: 20 Hz; power: 10 W; percent of illumination periods: 20% or 100%, n = 3–4). Right: Average device temperature for 12 hours (n = 3–4). (B) Left: ΔVO2 measured by the CLAMS for 2 hours in the μLED implanted and sham-operated mice receiving IP injection of norepinephrine (n = 6–7). The experiment was performed with adult male C57BL/6 mice. Right: AUC quantifications of ΔVO2 are shown. (C) Left: ΔVO2 measuring in littermate Opn3-KO mice receiving IP injection of norepinephrine (n = 3–4). Right: AUC quantifications are shown. (D) Pictures of surgery for in vivo illumination. Left and middle: Light device was placed on the top of the interscapular BAT (surrounded by yellow line). Right: Light was activated after the surgical recovery. Arrows indicate lighting. Data are represented as mean ± SEM. The p-values were determined as follows: (A) ordinary one-way ANOVA followed by Tukey’s multiple comparison test. **p < 0.01, ***p < 0.001; *significant difference between lighting and nonlighting. ([B] and [C], left) Two-way repeated measures ANOVA followed by Tukey’s multiple comparison test. ([B] and [C], right) Ordinary one-way ANOVA with Tukey’s multiple comparison test. *p < 0.05, **p < 0.01; *significant difference between light group and sham group. †p < 0.05, ††p < 0.01; †significant difference between light wavelength (white and red). The data for this figure can be found in the Dryad repository: https://doi.org/10.5061/dryad.p5hqbzkkv [70]. AUC, area under the curve; BAT, brown adipose tissue; CLAMS, Comprehensive Lab Animal Monitoring System; IP, intraperitoneal; KO, knockout; LED, light-emitting diode; Opn3, Opsin3; VO2, oxygen consumption. (TIF) [file pbio.3000630.s005.tif]

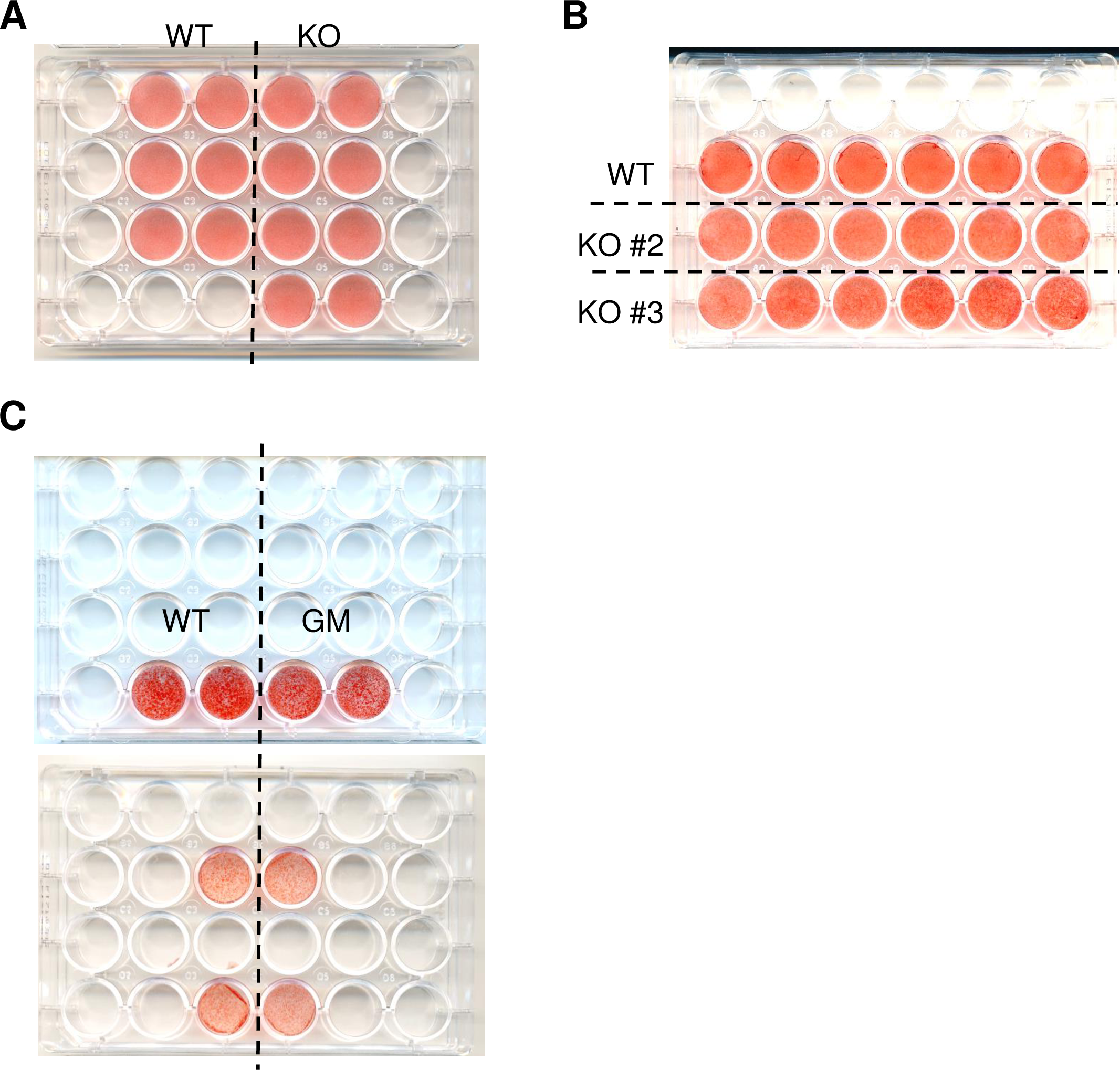

Supplement: S6 Fig — (TIF) [file pbio.3000630.s006.tif]

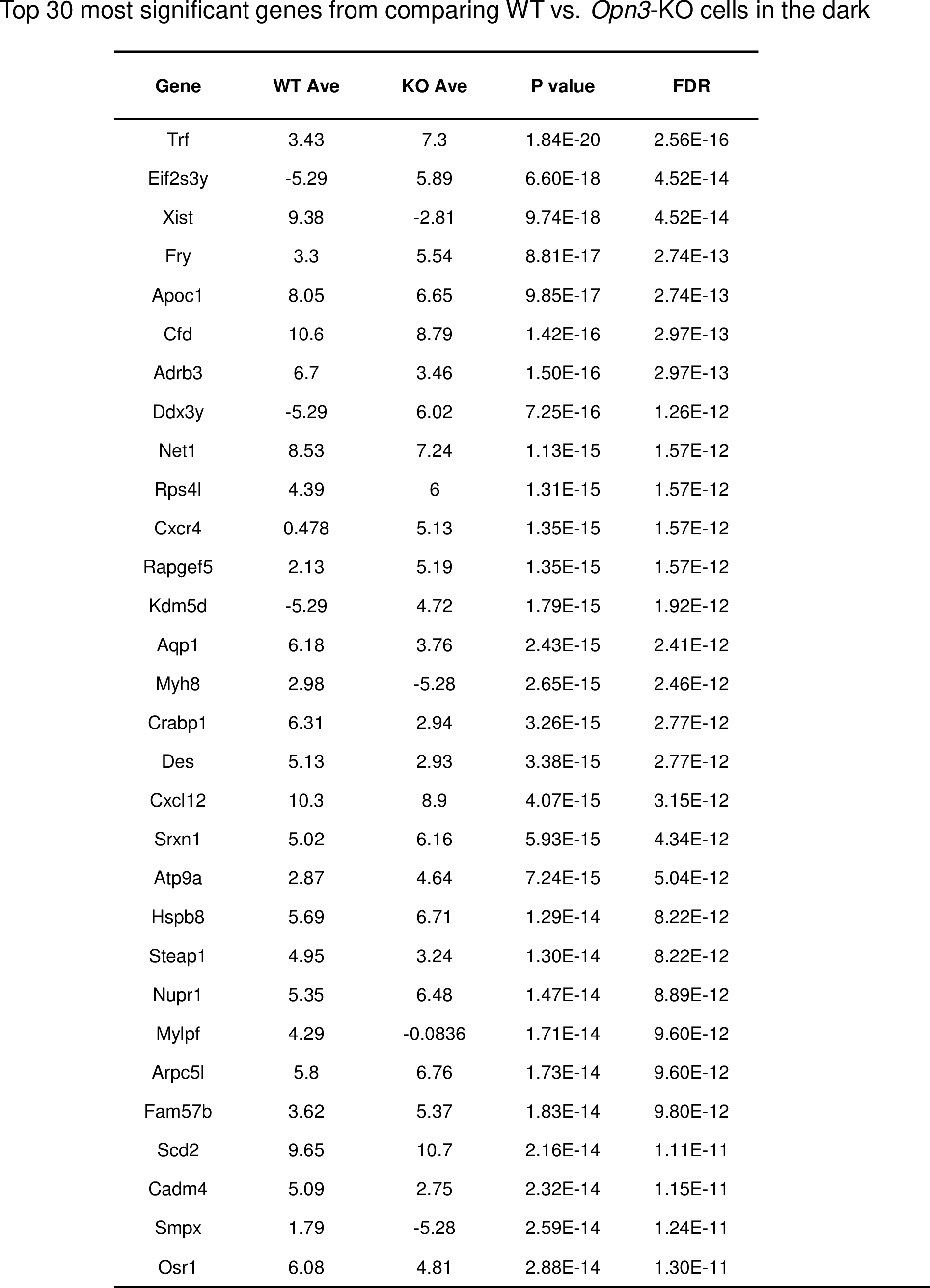

Supplement: S1 Table — KO, knockout; Opn3, Opsin3; WT, wild-type. (TIF) [file pbio.3000630.s007.tif]

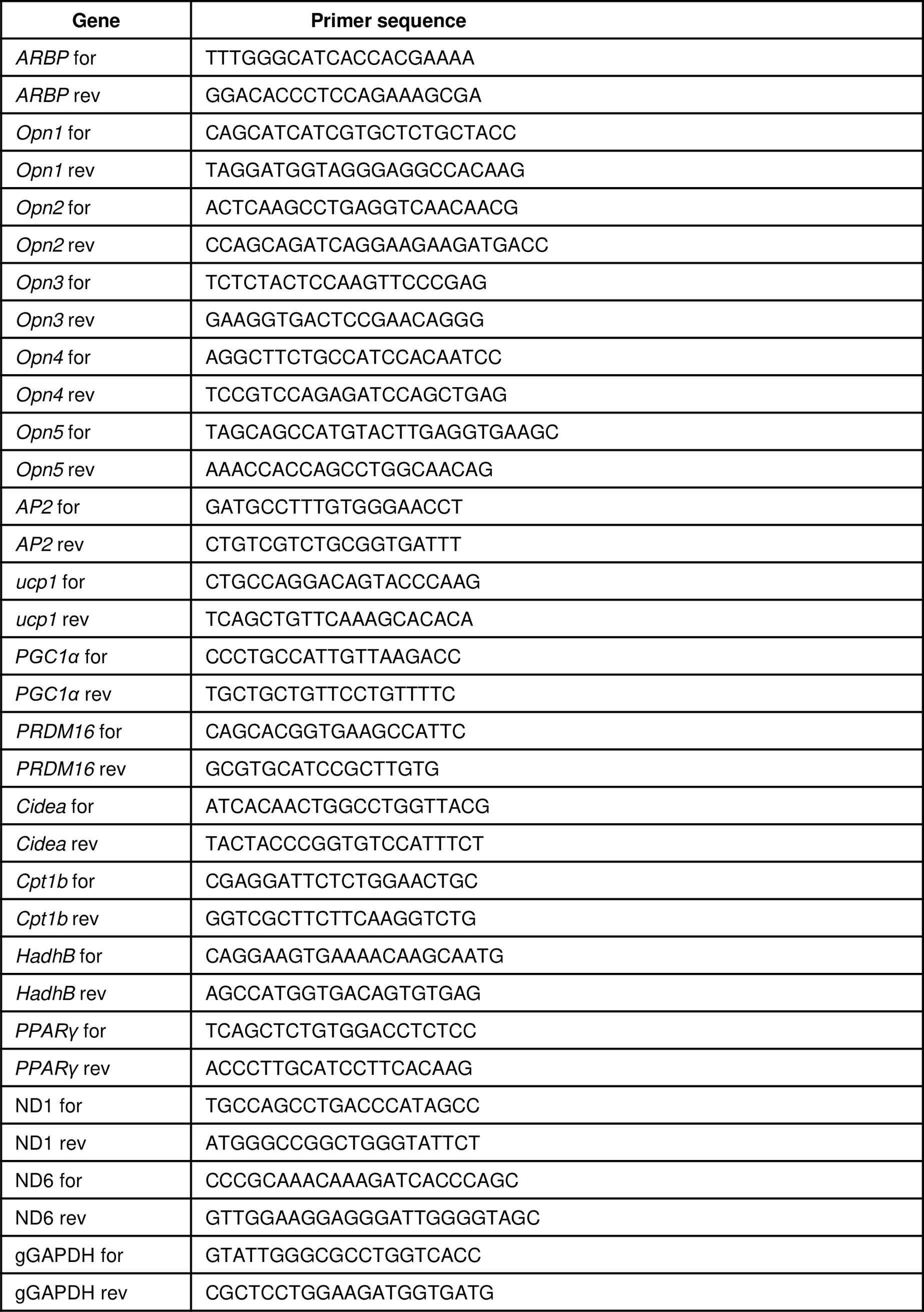

Supplement: S2 Table — (TIF) [file pbio.3000630.s008.tif]
