# Supplementary material for: Cell-autonomous light sensitivity via Opsin3 regulates fuel utilization in brown adipocytes
Source: PLoS Biol. 2020 Feb 10;18(2):e3000630. doi: 10.1371/journal.pbio.3000630 (PMC7034924; doi:10.1371/journal.pbio.3000630)

Fig 2C: Glut1 and GAPDH

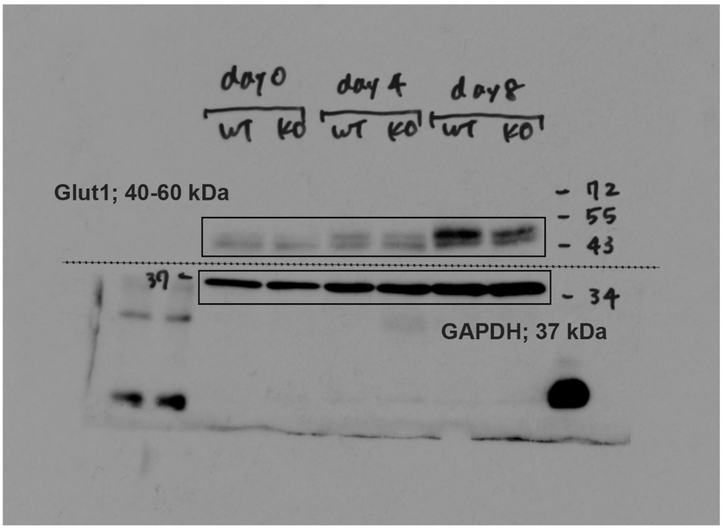

Fig 2F: Cpt1

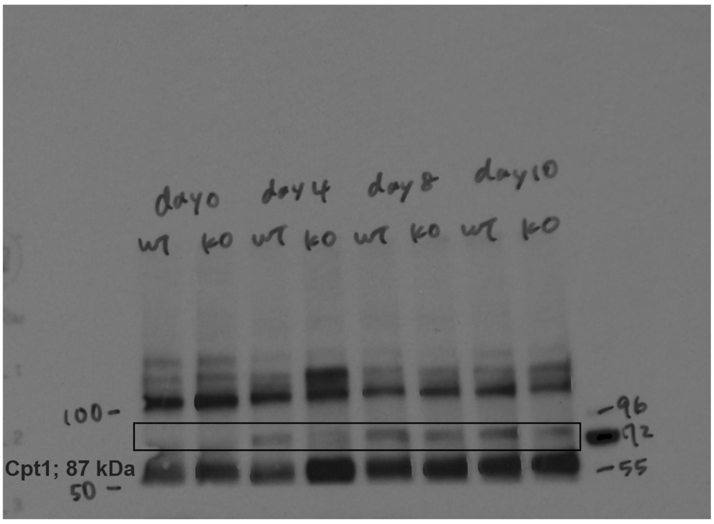

Fig 2F: GAPDH

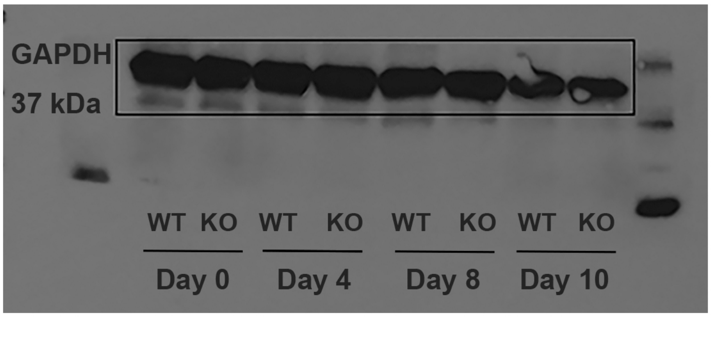

Fig 3F: CPT1

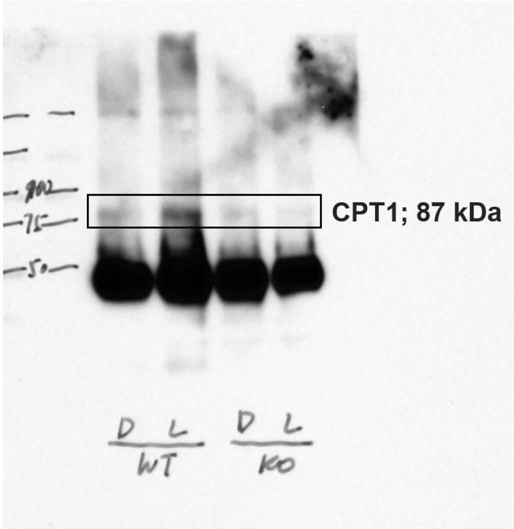

Fig 3F: b-tubulin

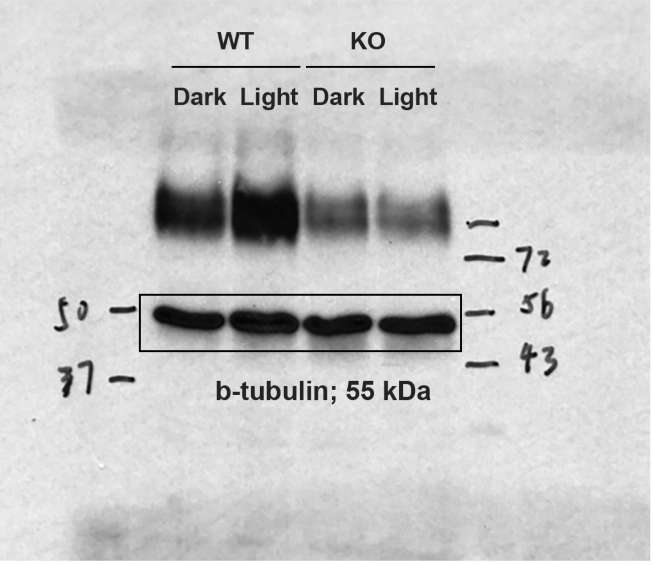

Fig 4C: CPT1

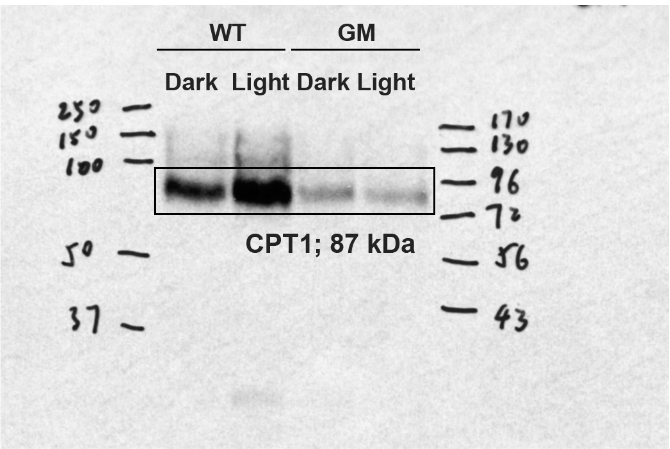

Fig 4C: b-tubulin

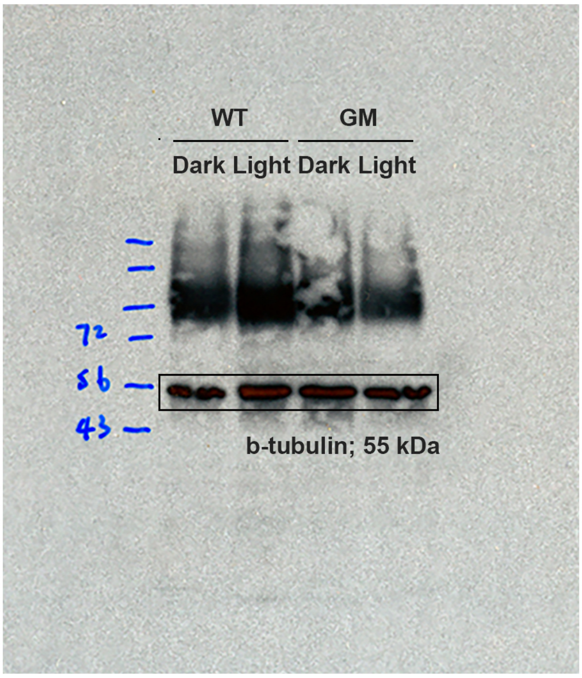

S2E Fig: AP2

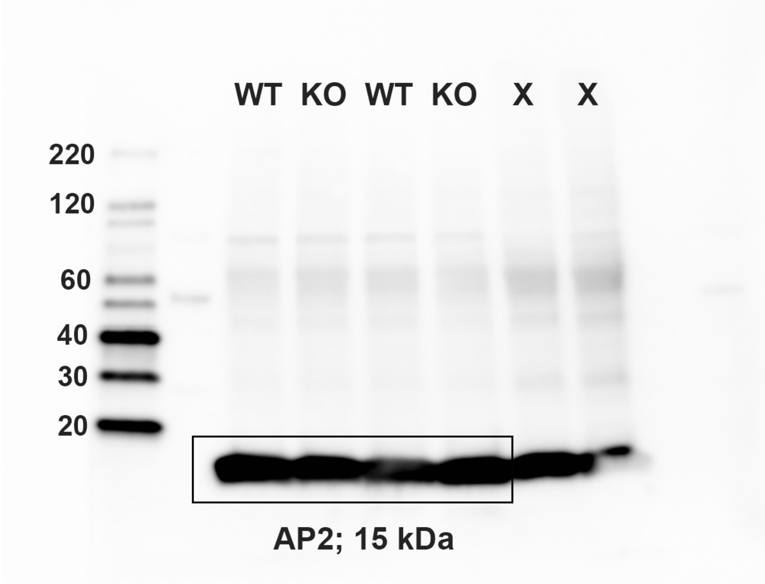

S2E Fig: GAPDH

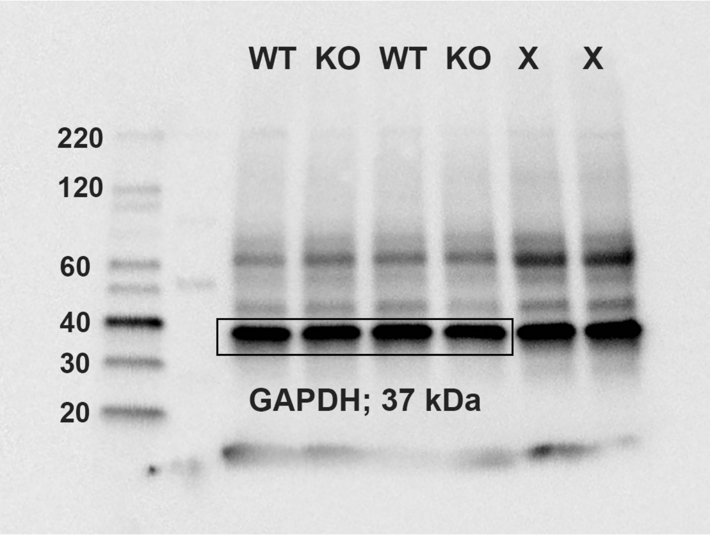

S2F Fig: PPARg

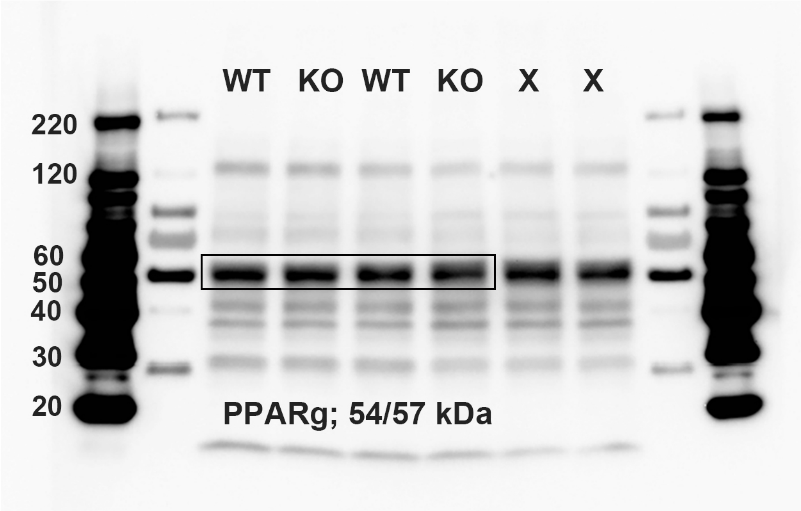

S2F Fig: GAPDH

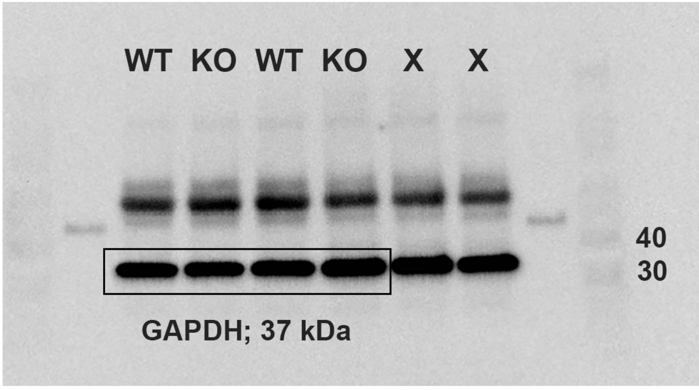

S2H Fig: Ucp1

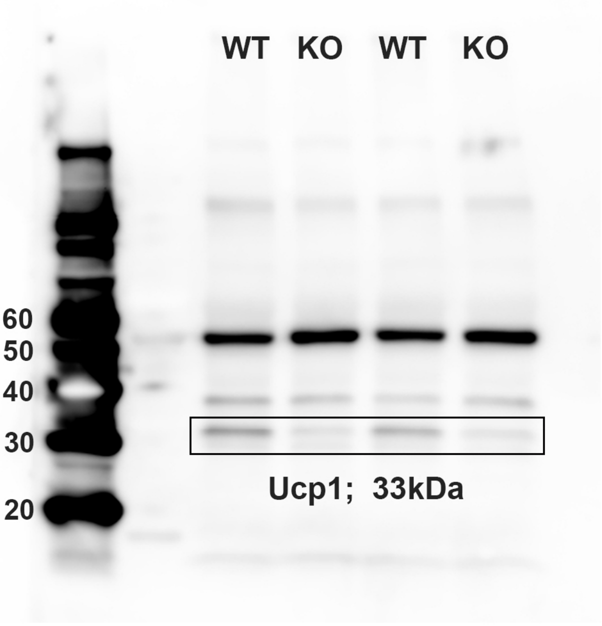

S2H Fig: GAPDH

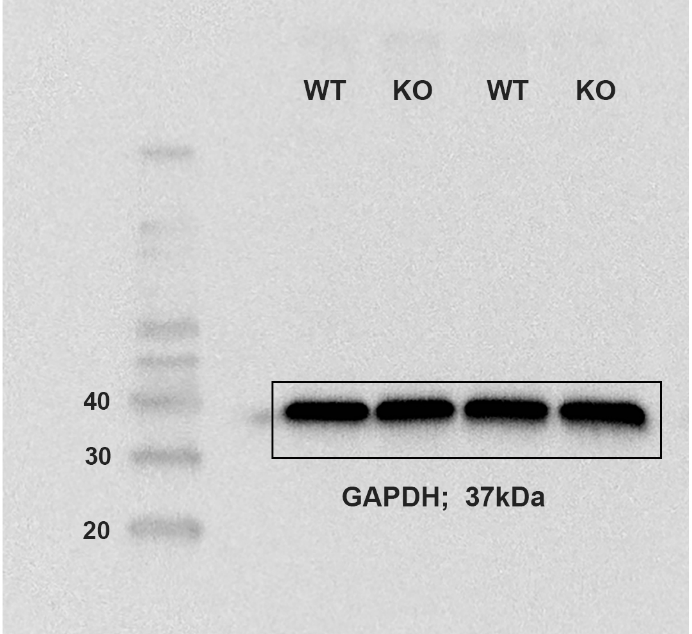

S2N Fig: HSL

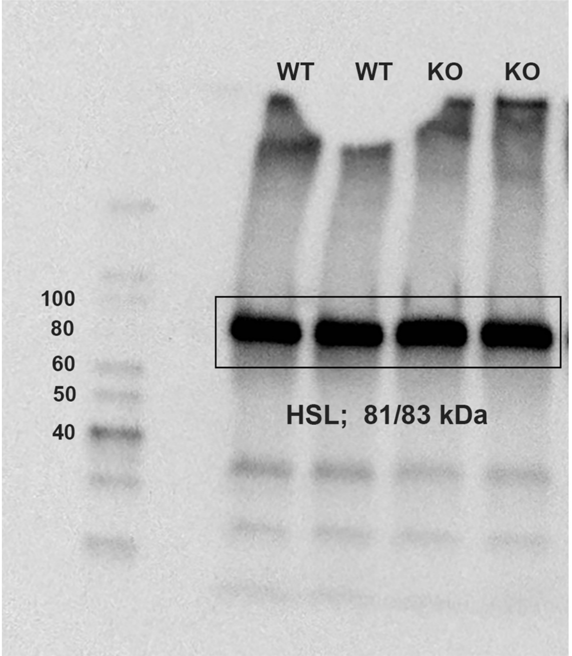

S2N Fig: pHSL (ser 563)

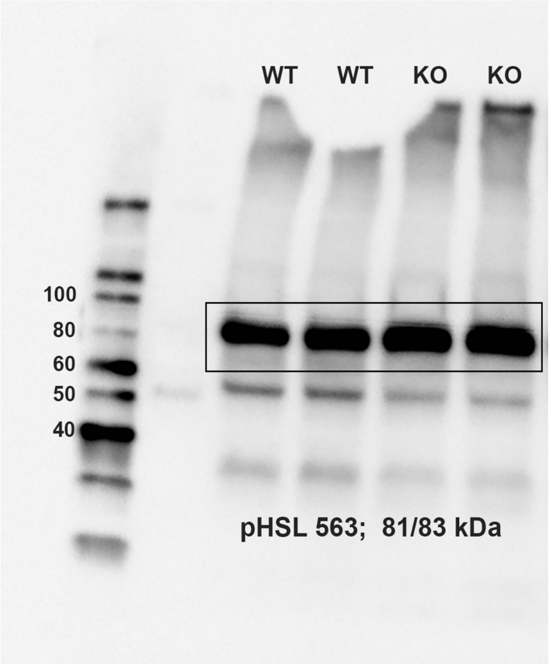

S2N Fig: pHSL (Ser 565)

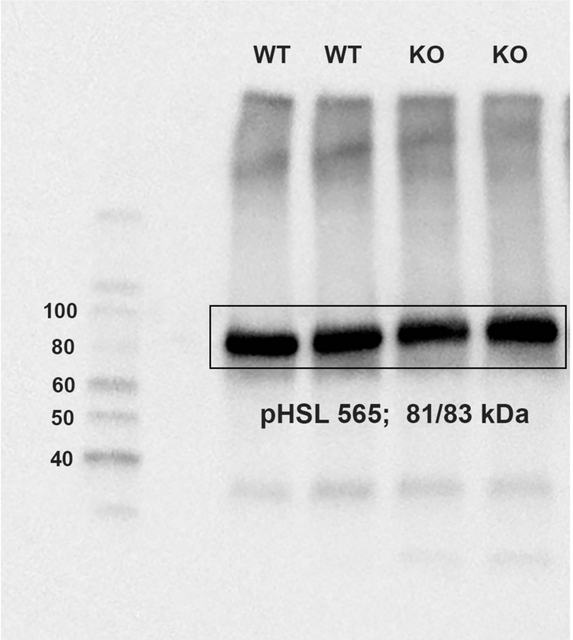

S2N Fig: pHSL (Ser 660)

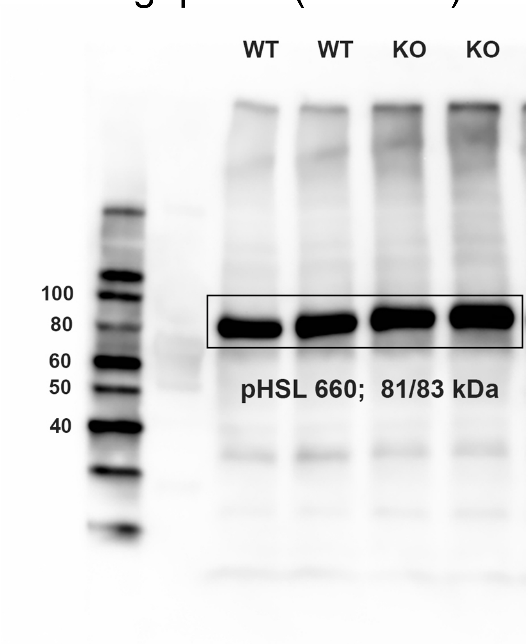

S2N Fig: ATGL

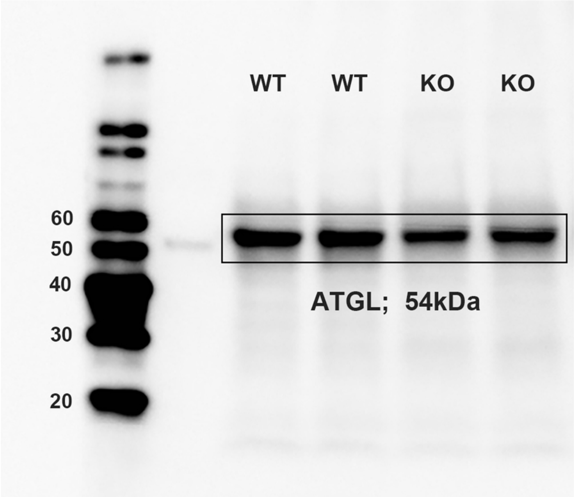

S2N Fig: GAPDH

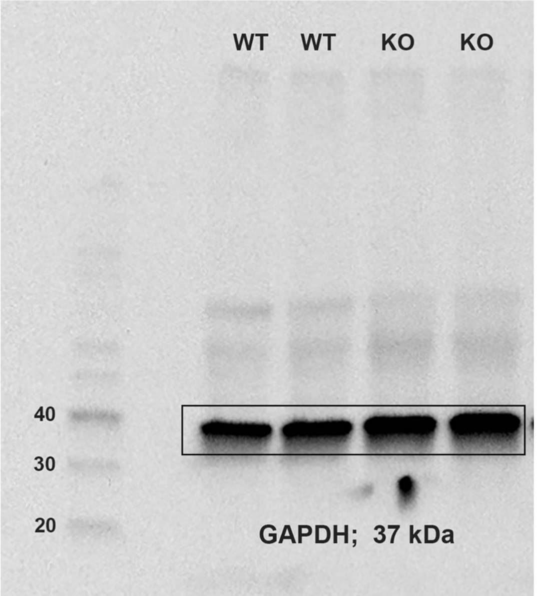

S3F Fig: ATGL

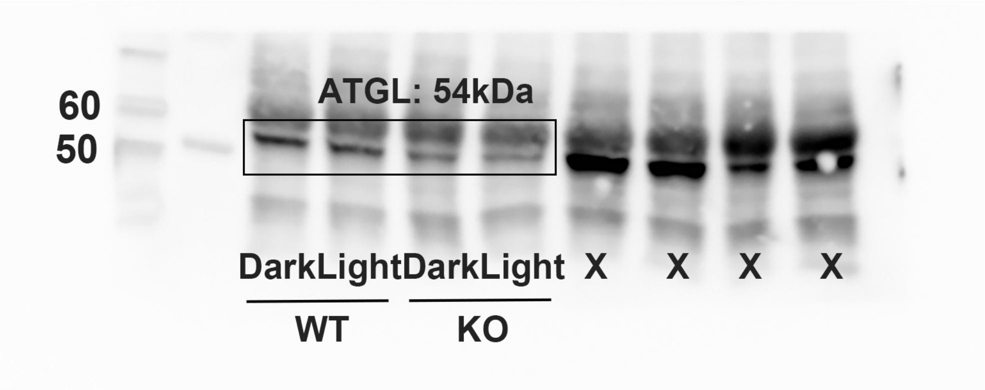

S3F Fig: GAPDH

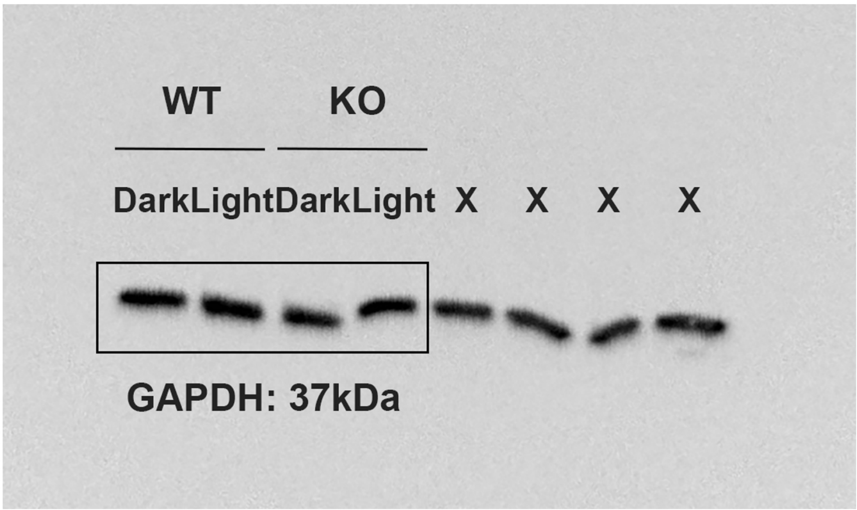

S4A Fig: AP2

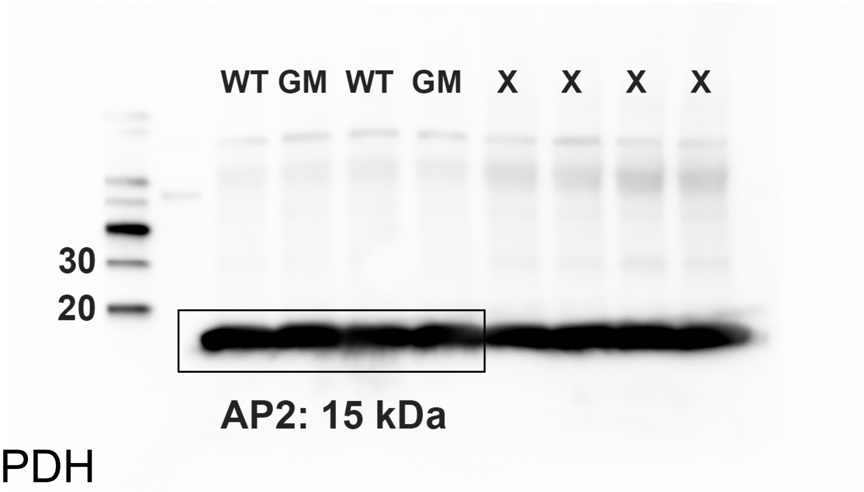

S4A Fig: GAPDH

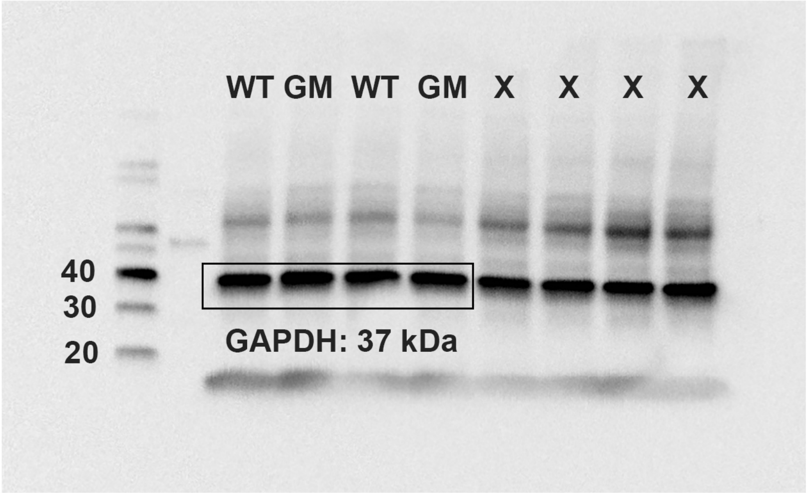

S4C Fig: Ucp1

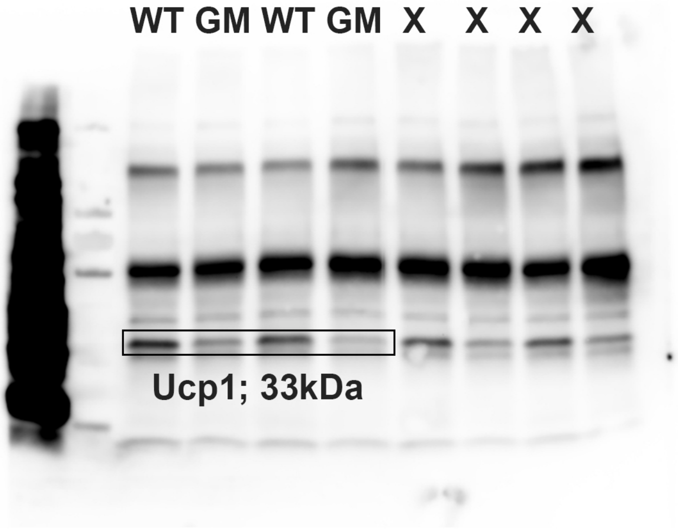

S4C Fig: GAPDH

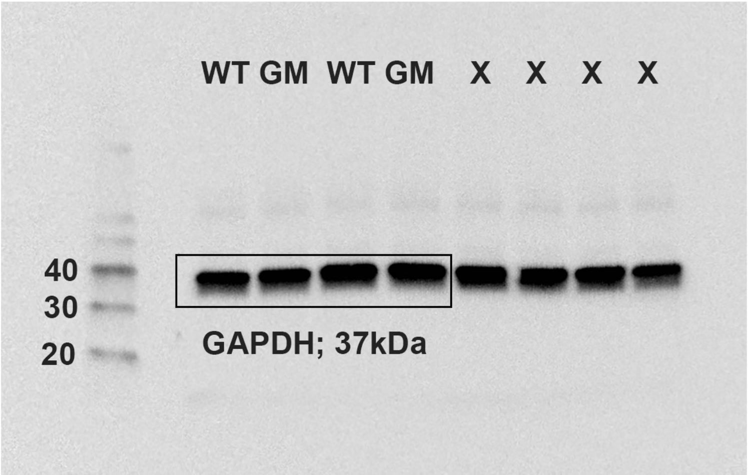

Supplement: S1 Raw Images — The data can be found in the Dryad repository: https://doi.org/10.5061/dryad.p5hqbzkkv [70]. (PDF) [file pbio.3000630.s009.pdf]
